# Supplementary material for: Binding kinetics drive G protein subtype selectivity at the β1-adrenergic receptor
Source: Nat Commun. 2024 Feb 13;15:1334. doi: 10.1038/s41467-024-45680-7 (PMC10864275; doi:10.1038/s41467-024-45680-7)
Supplement: Supplementary file 1 — Supplementary Information [file 41467_2024_45680_MOESM1_ESM.pdf]

Supplementary Information

Binding kinetics drive G protein subtype selectivity at the β_1-adrenergic receptor

Andrew J. Y. Jones[1,3], Thomas H. Harman[1,3], Matthew Harris[2], Oliver E. Lewis[1], Graham Ladds[2], Daniel Nietlispach[1*]

[1] Department of Biochemistry, University of Cambridge, 80 Tennis Court Road, Cambridge CB2 1GA, UK.
[2] Department of Pharmacology, University of Cambridge, Tennis Court Road, Cambridge CB2 1PD.
[3] These authors contributed equally.

*To whom correspondence may be addressed: Email dn206@cam.ac.uk

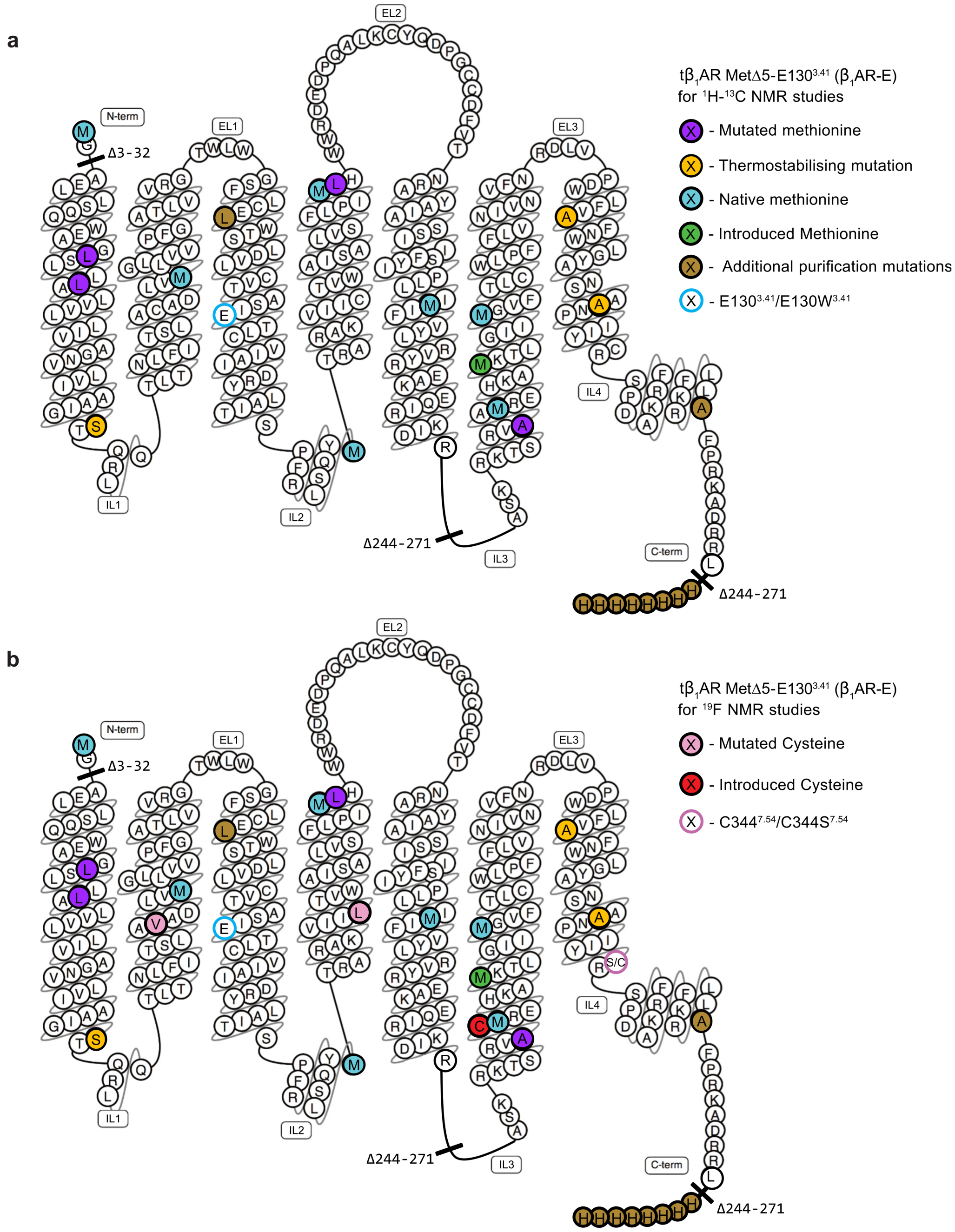

Supplementary Figure 1. Snake diagrams of the β_1AR constructs. a) The β_1AR construct used in ^{1}H-^{13}C HMQC NMR experiments and BLI assays. Intracellular loop (IL) and extracellular loop (EL) regions are indicated. All mutations are relative to the native turkey β_1AR sequence. β_1AR-E and β_1AR-W differed by mutation of position $130^{3.41}$, shown with a light blue ring in TM3. ^{1}H-^{13}C NMR studies of β_1AR-E and β_1AR-W used constructs with the mutated methionine residues (purple), thermostabilising mutations (orange), and purification mutations (brown). Native methionine probes are shown in blue. NMR experiments using L289M$^{6.34}$ had the introduced methionine mutation (green) present. BLI studies of β_1AR-E and β_1AR-W utilised the same constructs as ^{1}H-^{13}C NMR studies with an additional N-terminal avi-tag (GLNDIFEAQKIEWHE) between residues M1 and G2. **b)** The β_1AR construct used in ^{19}F NMR experiments. The constructs were the same as the β_1AR-E and β_1AR-W constructs used in ^{1}H-^{13}C NMR studies with additional mutations for ^{19}F labelling. TM7 labelling (^{TET}C344$^{7.54}$) used the mutated cysteines (pink) and native C344$^{7.54}$ (pink ring). TM6 labelling (A282C$^{BTFA,6.27}$) used the mutated cysteines, introduced cysteine (red), and mutated C344S$^{7.54}$ (pink ring). See methods for details of residue positions and explanations for mutations shown in **a)** and **b).**

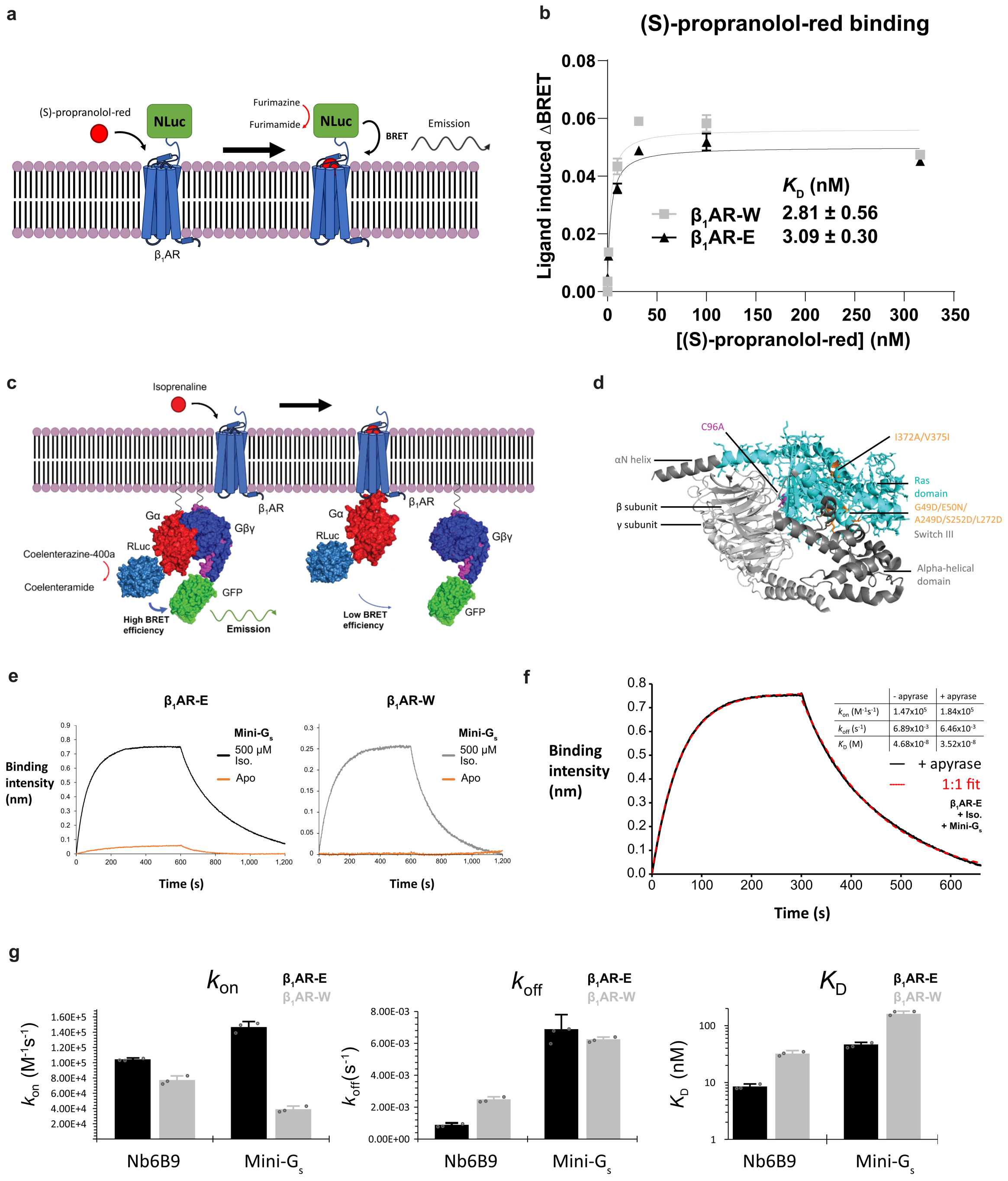

Supplementary Figure 2. Schemes for *in cell* validation of β_1AR NMR constructs, and validation of *in vitro* mini-G protein binding. a) A scheme for the luminescence-based ligand binding assay for determination of ligand K_i to β_1AR. N-Luciferase (NLuc) tagged β_1AR binds to (S)-propranolol-red[1]. Catalysis of furimazine to furimamide by NLuc generates luminescence which is transferred by BRET to (S)-propranolol-red and emitted. Titration with non-BRET active ligand displaces (S)-propranolol-red and reduces the BRET signal, facilitating binding curve generation and K_i calculations[2]. **b)** (S)-propranolol binding to the β_1AR constructs. K_D values are shown in the inset. **c)** A scheme of the TRUPATH assay[3]. Gα and Gβγ proteins are labelled with R-Luc8 and GFP2, respectively. Binding of ligand to receptor induces receptor activation and G protein binding. Dissociation of the Gα and Gβγ subunits reduces the BRET efficiency, therefore reducing emission intensity. Titration of ligand facilitates generation of EC_{50} curves. **d)** A structural comparison of $G\alpha_s$ (PDB: 6EG8)[4] with mini-G_s protein[5] showing conserved regions, deletions, and mutations. Cyan, conserved sequence, grey, deleted sequence, orange, point mutations, magenta, C96A mutation, white, Gβγ subunit. **e)** BLI binding curves showing the ligand dependence

of mini-G_s binding to β_1AR constructs. Left, β_1AR-E (black, 500 μM isoprenaline (Iso.), orange, apo (no ligand)), right, β_1AR-W (grey, 500 μM isoprenaline, orange, apo (no ligand)). The mini-G_s concentration used was 2 μM. **f)** BLI binding curves showing the effect of apyrase incubation on β_1AR-E binding to mini-G_s in the presence of isoprenaline. Black solid line, mini-G_s binding following incubation with apyrase to remove bound GDP (overnight), red dotted line, 1:1 interaction stoichiometry model curve. Inset, equivalent kinetic data for binding curve compared to conditions without apyrase. **g)** BLI kinetic data for binding of isoprenaline-bound β_1AR to Nb6B9, compared with binding to mini-G_s. Black, β_1AR-E, grey, β_1AR-W. Values were the average of triplicates (n=3 individual repeats). Error bars represent ± one standard deviation. Note the logarithmic axis for K_D data.

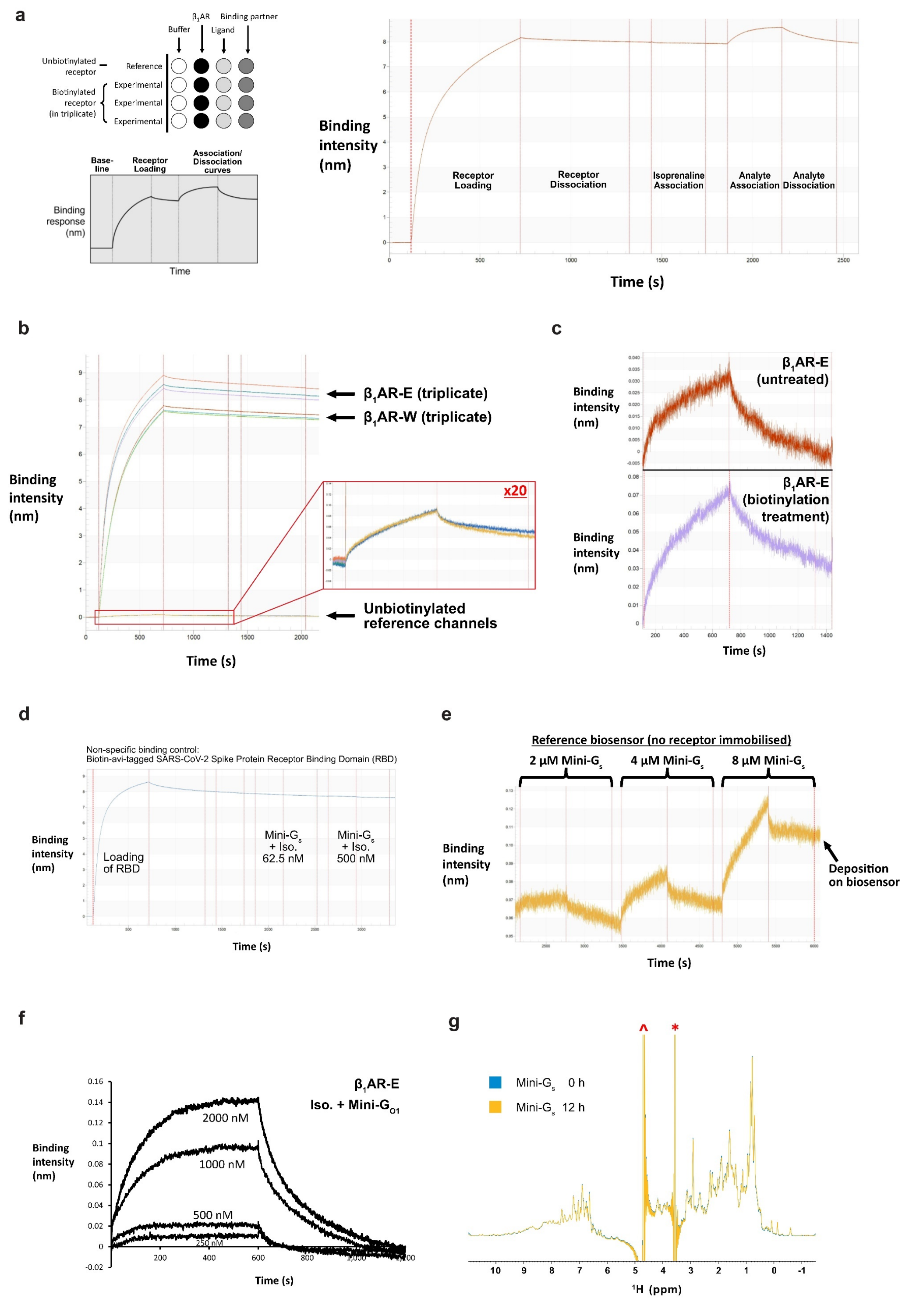

Supplementary Figure 3. Controls for β_1AR-E BLI assays. a) A scheme and data to show BLI protocols. Left, a scheme showing the BLI experiment and wells. See methods for details. Right, data showing an example of loading of β_1AR-E and generation of binding curves with isoprenaline and mini-G_S. The solid red line shows the binding response. Dotted vertical red lines separate each step. Note the dissociation step after receptor loading indicates biotinylated receptor is specifically immobilised onto the streptavidin biosensors. **b)** An example of biotinylated avi-β_1AR-E and avi-β_1AR-W loading onto streptavidin (SA) biosensors in triplicate. The inset boxed in red (y-axis enlarged 20x) demonstrates association of the unbiotinylated receptor controls is minimal, indicating biotin-specific loading onto the biosensors. **c)** Loading controls and biotinylation specificity controls for β_1AR-E shows avi-tag specific biotinylation and minimal non-specific loading onto SA biosensors. Top, β_1AR-E without the avi-tag and without biotinylation treatment. Bottom, β_1AR-E without the avi-tag and with biotinylation treatment. **d)** Mini-G_S shows no binding to unrelated protein SARS-CoV2 Spike protein Receptor Binding

Domain (RBD). Blue, BLI trace showing loading of an avi-tagged and biotinylated RBD protein (300 nM) onto SA tips in β_1AR BLI buffer, and association and dissociation steps assessing mini-G_s (62.5 and 500 nM) association, with isoprenaline (500 µM). There is negligible interaction between the ligand and analyte, supporting a specific interaction between β_1AR and mini-G_s. **e)** Concentration range determination. The reference biosensor (unbiotinylated avi-β_1AR-E in the loading step) was dipped into wells containing mini-G analytes. Deposition of analyte on the biosensor is clearly visible at 8 µM, limiting the concentration range for BLI experiments. Shown is an example for mini-G_s. **f)** BLI dose-response trial for mini-G_{o1} binding to β_1AR-E in the presence of 500 µM isoprenaline. The binding response is too small to reliably calculate binding affinities below 1000 nM. **g)** ^{1}H NMR assessment of analyte stability. Shown is a representative experiment with mini-G_s. Protein was incubated at 298 K for 12 h, and 1D ^{1}H NMR experiments were recorded at 0 h (blue) and 12 h (orange) time points. ^ indicates suppressed water signal, * indicates TrisHCl buffer signal.

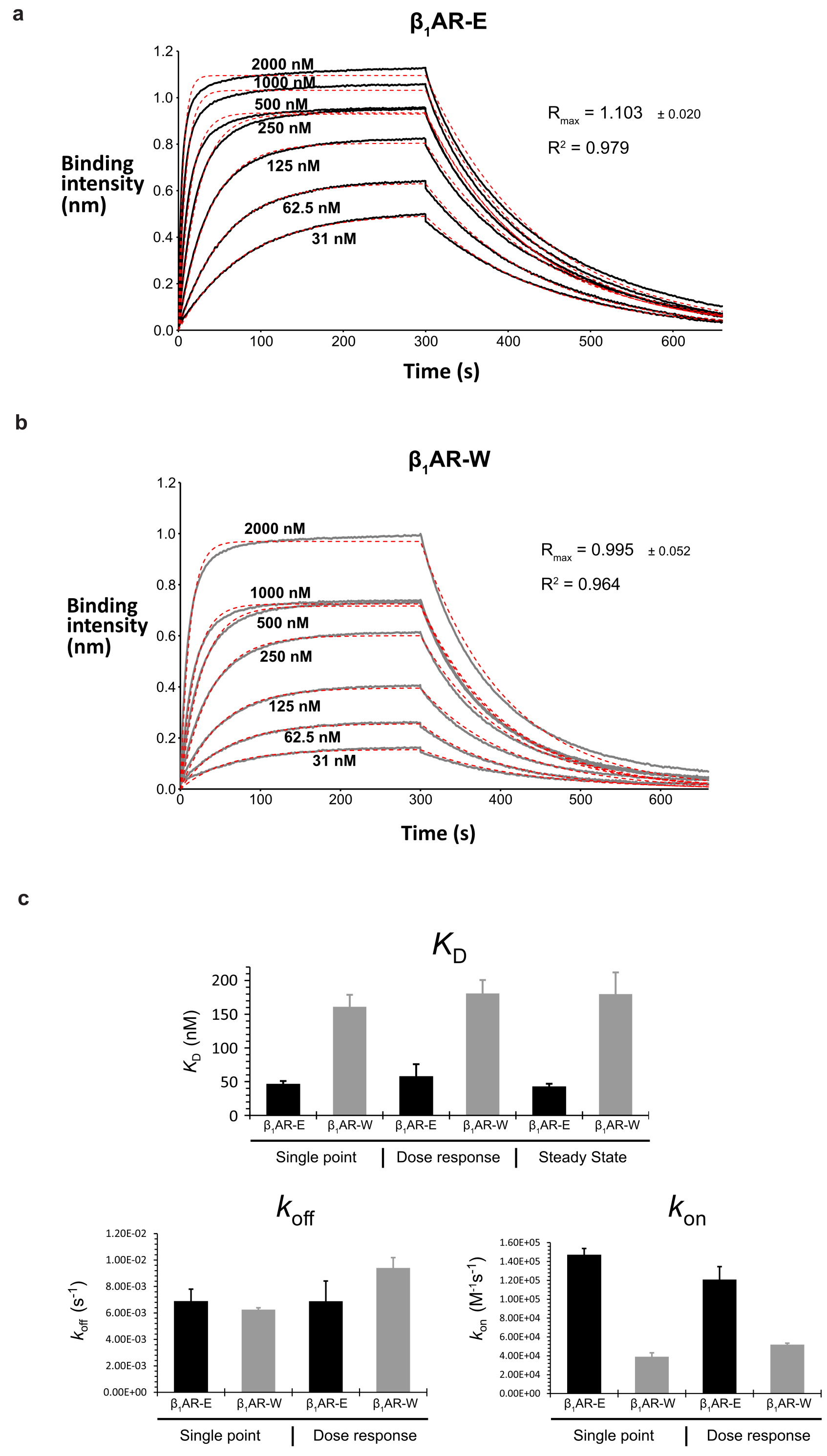

Supplementary Figure 4. Dose-response BLI experiments for β₁AR binding to mini-Gs. a) A BLI dose-response experiment for β₁AR-E binding to mini-Gs in the presence of isoprenaline. Raw data is shown as a solid black line, and 1:1 global fitting with R_{max} unlinked by sensors are shown in dashed red lines. Concentrations of mini-Gs are shown on the isotherms. The isoprenaline concentration was 500 μM. The curves were used to calculate K_D values using the 1:1 global fitting or by steady state response analysis. Steady state response curves were used to calculate R_{max} values, shown on the right with corresponding R^2 value. **b)** A BLI dose-response experiment for β₁AR-W binding to mini-Gs in the presence of isoprenaline. Details are as described for **a)**. For **a)** and **b)**, note the differences in R_{max} between the two constructs may relate to the slightly smaller loading of β₁AR-W relative to β₁AR-E (Supplementary Fig. 3b). **c)** A comparison of K_D, k_{on}, and

k_{off} values calculated from optimised single concentration isotherms, dose-response curves with global fitting, and steady state response curves for β_1AR-E and β_1AR-W binding to mini-G_s in the presence of 500 μM isoprenaline. Values for the single point measurements are the mean of triplicates (n=3 individual repeats), and error bars show SD for these replicates. Values for the dose response and steady state measurements were calculated using the data shown in **a** and **b**, and error bars show calculated errors from Data Analysis V 11.0.0.4 (Pall ForteBio LLC) software. See Methods and Supplementary Methods for details on the fitting parameters.

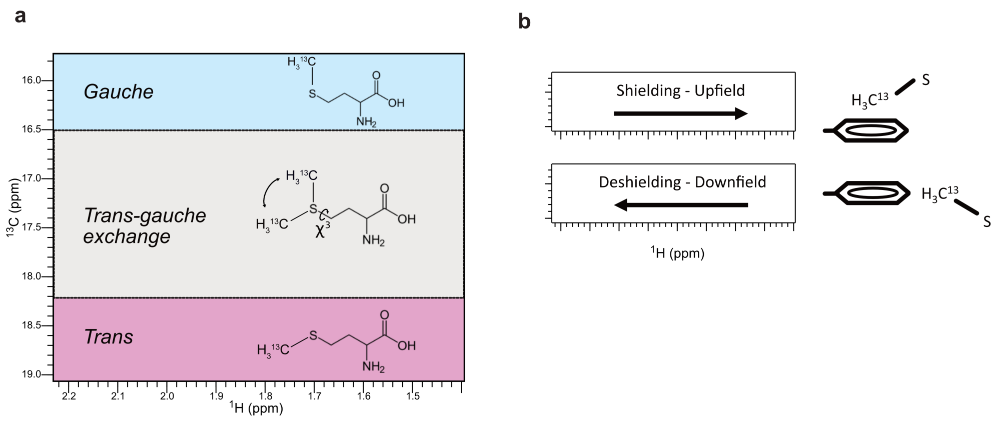

Supplementary Figure 5. The effect of ^{13}C-methyl methionine χ^3 conformers on methyl ^{13}C and ^{1}H chemical shifts. a) The effect of χ^3 conformers on ^{13}C shifts. Trans and gauche χ^3 conformers have defined ^{13}C shift regions (red and blue, respectively), and ^{13}C chemical shift values between these regions represent χ^3 sampling both trans and gauche conformers in conformational exchange (grey). Methyl ^{13}C chemical shifts have been shown to be a suitable reporter of conformational changes related to receptor activation[6,7]. **b)** The effect of ring currents of aromatic groups on ^{1}H chemical shifts of methyl group probes. Top, a face-on orientation of the aromatic system to the methyl probe affects ring current contributions to ^{1}H shifts through shielding of the protons, causing an upfield shift. Bottom, an edge-on orientation has the converse effect, deshielding protons and causing a downfield shift[8].

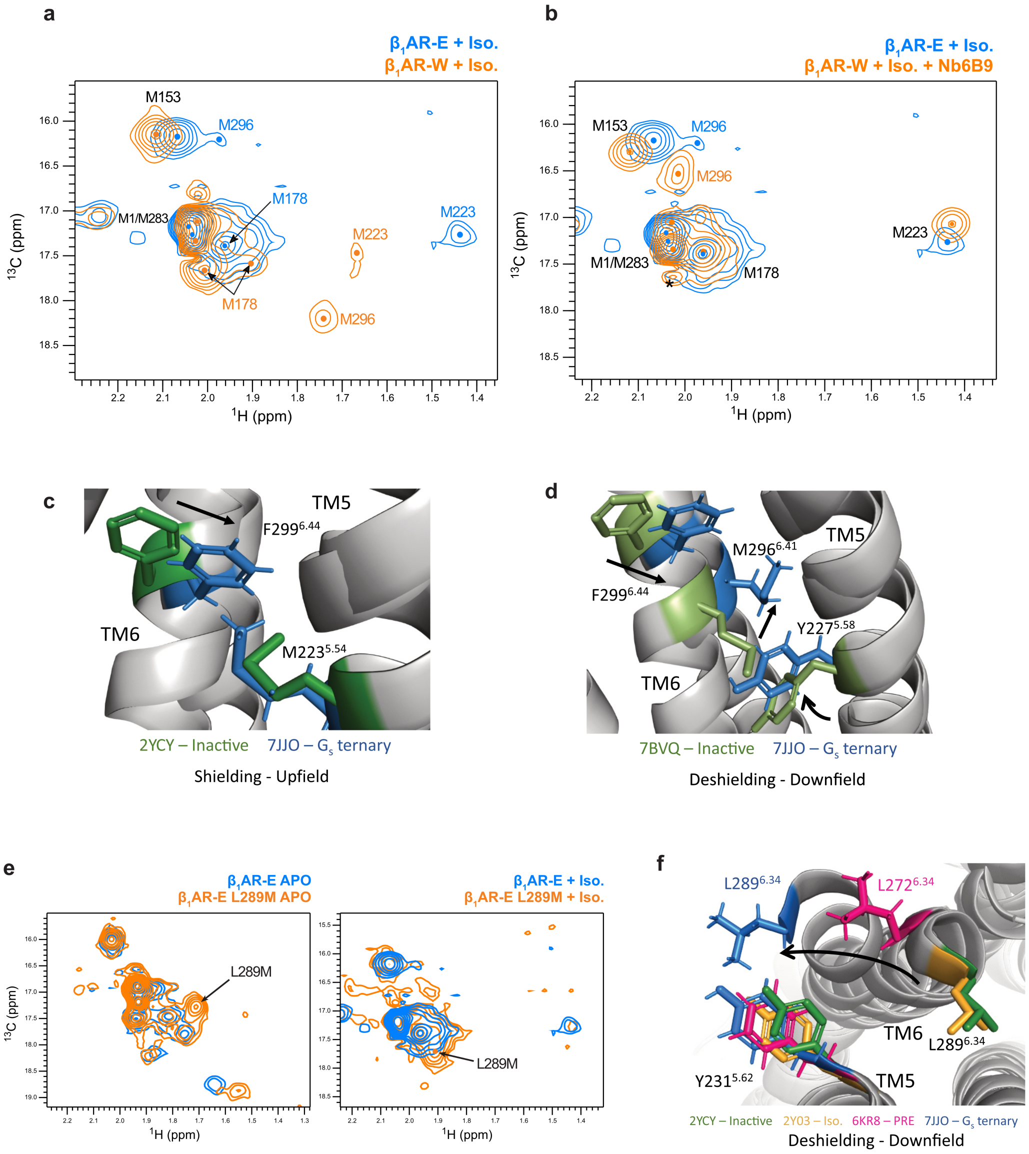

Supplementary Figure 6. Isoprenaline addition to β_1AR-E results in population of an active state conformation similar to ternary complexes. a) An overlay of the ^{1}H-^{13}C HMQC NMR spectra of isoprenaline-bound (Iso.) β_1AR-E and isoprenaline-bound β_1AR-W, recorded at 308 K. **b)** An overlay of the ^{1}H-^{13}C HMQC NMR spectra of isoprenaline-bound β_1AR-E and isoprenaline-bound β_1AR-W with 2 molar equivalents of Nb6B9, recorded at 308 K. * indicates an artifact related to ^{1}H 180˚ pulse imperfection. **c)** A comparison of structures of the inactive cyanopindolol-bound β_1AR crystal structure (green - PDB: 2YCY)[9] with the isoprenaline-bound β_1AR-G$_s$ ternary cryo-EM structure (blue - PDB: 7JJO)[10] showing the conformation of aromatic residue F299$^{6.44}$ and methyl probe M223$^{5.54}$. The benzyl ring of F299$^{6.44}$ in the active receptor (blue) adopts a face-on position above M223$^{5.54}$ that would exert a strongly shielding ring current shift. **d)** A comparison of the inactive carazolol-bound human β_1AR crystal structure (light green - PDB: 6BVQ)[11] with the isoprenaline-bound β_1AR-G$_s$ ternary cryo-EM structure (PDB: 7JJO)[10] showing the conformations of aromatic residues around methyl probe M296$^{6.41}$. Y227$^{5.58}$ is absent from the inactive cyanopindolol-bound β_1AR structure[9] hence the use of an alternative inactive structure. Both Y227$^{5.58}$ and F299$^{6.44}$ adopt side-on orientations with respect to M296$^{6.41}$ which would result in a strongly deshielding effect from the altered ring current contributions. **e)** An overlay of the ^{1}H-^{13}C HMQC NMR spectra of apo β_1AR-E L289$^{6.34}$ and apo β_1AR-E L289M$^{6.34}$ (left), and of isoprenaline-bound (Iso.) β_1AR-E L289$^{6.34}$ and isoprenaline-bound β_1AR-E L289M$^{6.34}$ (right), recorded at 308 K. Experimental conditions were identical between the two experiments to facilitate assignment of L289M$^{6.34}$. **f)** A comparison of the inactive and active βAR structures showing the distance from aromatic residue Y231$^{5.62}$ to L$^{6.34}$ (L289M$^{6.34}$ in our data). Structures: inactive cyanopindolol-bound β_1AR crystal structure (green - PDB: 2YCY)[9], isoprenaline-bound β_1AR crystal structure (orange - PDB: 2Y03)[12], isoprenaline-bound β_2AR PRE-based

structure (Pink - PDB: 6KR8)[13], and isoprenaline-bound β_1AR-G_s ternary cryo-EM structure (blue - PDB: 7JJO)[10]. $L^{6.34}$ retains an edge-on orientation with $Y231^{5.62}$, thus the ring current from this orientation would result in a downfield ^1H shift which increases in magnitude with proximity.

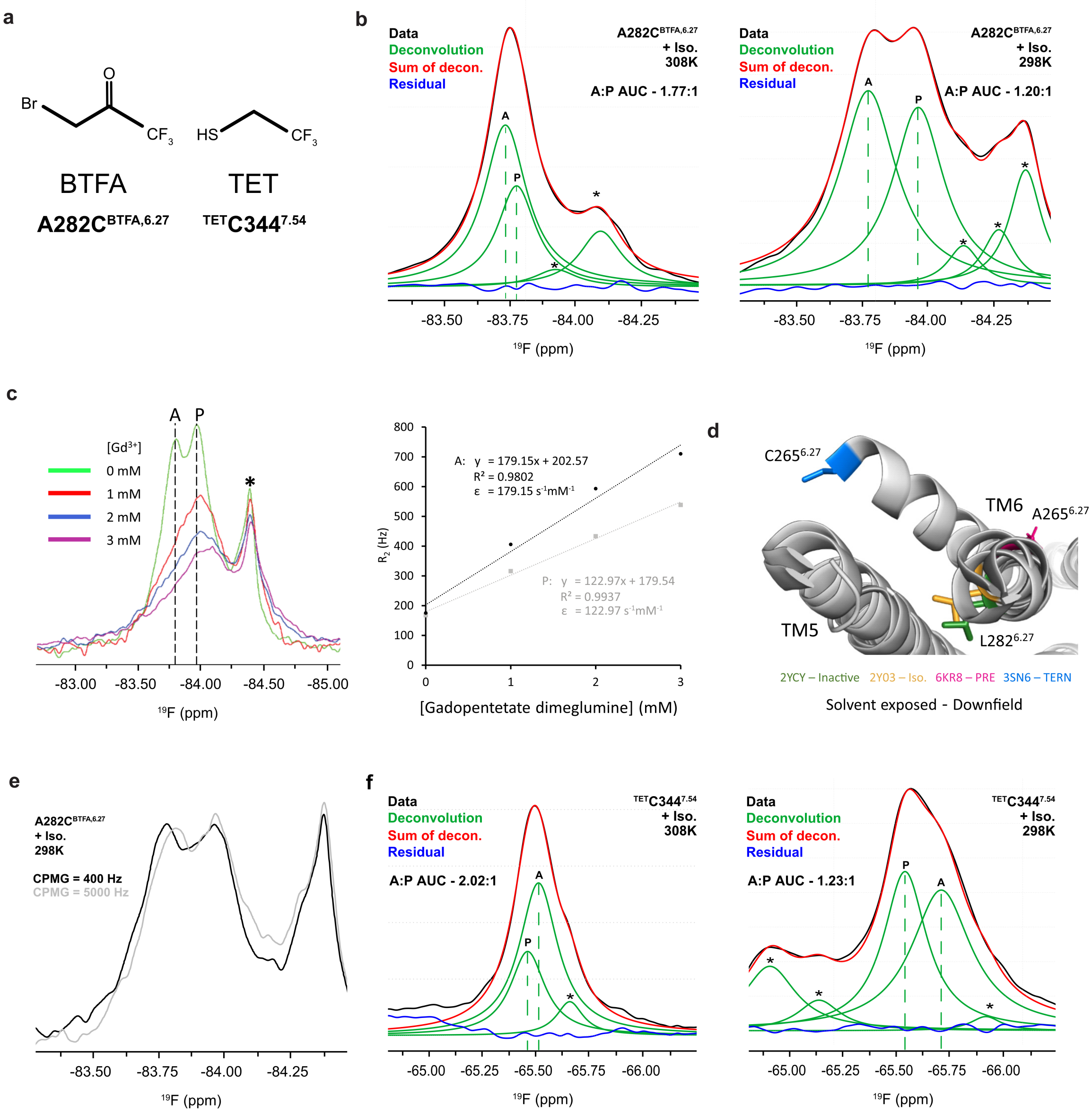

Supplementary Figure 7. ^{19}F NMR spectra show isoprenaline-bound β_1AR-E occupies the active and pre-active states. a) The ^{19}F probes used in this study. BTFA forms stable C-S bonds. TET forms S-S disulphide bonds, which are more susceptible to hydrolysis. **b)** Deconvolution of A282C$^{BTFA,6.27}$ ^{19}F NMR spectra recorded in the presence of 1 mM isoprenaline (Iso.) at 308 K (left) or 298 K (right). The chemical shifts of active state (A) and pre-active state (P) are indicated with dashed green lines. The residual (blue) is the subtraction of the sum of the simulated peaks (Sum of decon., red) from the raw data (black). Individual simulated peaks are shown in green. * indicates known degradation products. The ratio of the area under the curve (AUC) for the simulated active and pre-active states is shown. **c)** Solvent PRE experiments examining A282C$^{BTFA,6.27}$ solvent accessibility. Left, reference spectra for a titration series of Gd^{3+} into samples with isoprenaline-bound A282C$^{BTFA,6.27}$ at 298 K. The active (A) and pre-active (P) states are indicated. * indicates a degradation product. Right, a plot of R_2 relaxation rate against Gd^{3+} concentration. R_2 rates were calculated using a two-point relaxation measurement (see Methods). Linear fits and associated R^2 values are shown for the active (A, black) and pre-active (P, grey) states. The relaxation enhancement values (ε) due to Gd^{3+} addition are the gradient of the linear fits. **d)** A comparison of structures showing changes around the A282C$^{BTFA,6.27}$ probe. Structures: inactive cyanopindolol-bound β_1AR crystal structure (green - PDB: 2YCY)[9], isoprenaline-bound β_1AR crystal structure (orange - PDB: 2Y03)[12], isoprenaline-bound β_2AR PRE-based structure (Pink - PDB: 6KR8)[13], isoprenaline-bound β_2AR-G_s complex crystal structure (Blue – PDB: 3SN6)[14]. L282$^{6.27}$ moves from a less solvent exposed position behind TM5 to a solvent exposed position (A265$^{6.27}$ in β_2AR) through TM6 rotation. A282C$^{6.27}$ is unresolved in the β_1AR-G_s ternary complex structure (PDB: 7JJO)[10]. **e)** Relaxation

dispersion (CPMG) experiments of isoprenaline-bound A282C$^{BTFA,6.27}$ at 298 K with fast pulsing (5000 Hz, grey) and slow pulsing (400 Hz, black). See methods. **f)** Deconvolution of isoprenaline-bound ^{TET}C344$^{7.54}$ ^{19}F NMR spectra recorded at 308 K (left) or 298 K (right). Symbols are as in **b)**.

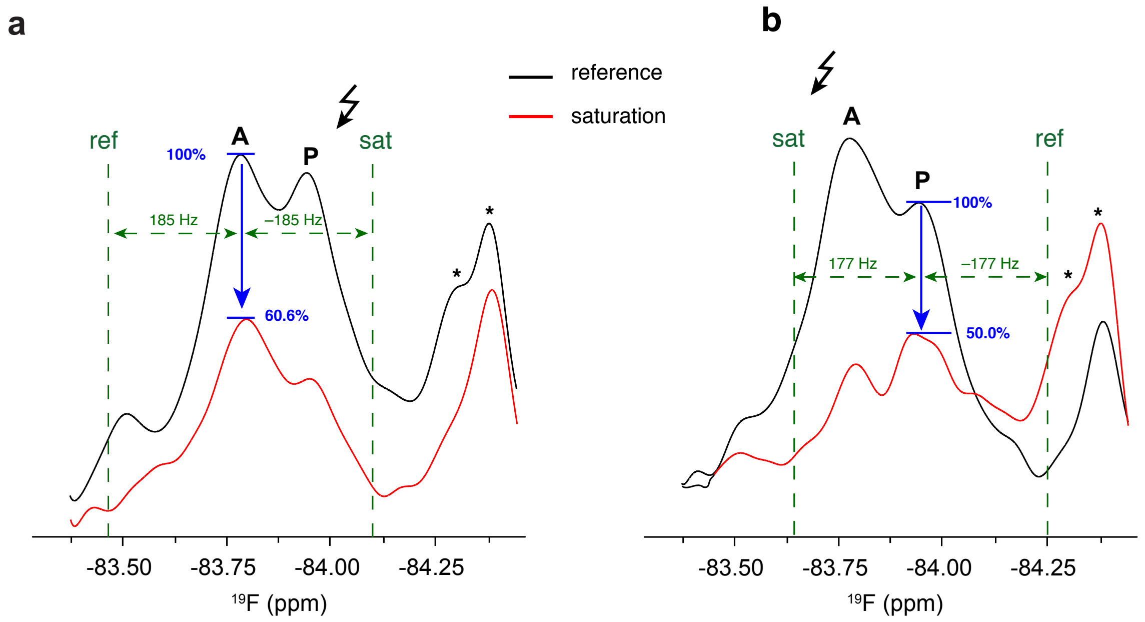

Supplementary Figure 8. Sub-second conformational exchange of β$_1$AR-E A282C$^{BTFA,6.27}$ explored by ^{19}F NMR saturation transfer. Pairwise 1D ^{19}F NMR experiments were recorded at 298 K following irradiation with a weak RF field (irradiation time of 1 s, saturation field strength 25 Hz) applied at either of the two offset positions located symmetrically (dashed green lines with the corresponding offset positions of the saturation field indicated) around the peak of interest, which resulted in a saturation spectrum (red, irradiation at position 'sat') or a reference spectrum (black, irradiation at position 'ref'), respectively. At the corresponding position of the peak of interest, the reduction in signal intensity due to saturation transfer is indicated by a blue arrow, with the signal intensity in the saturation spectrum given as a percentage of the intensity in the reference spectrum. **a)** β$_1$AR-E A282C$^{BTFA,6.27}$ bound to isoprenaline. The signal of the active state (A) shows attenuation upon irradiation of the pre-active state (P), indicative of conformational interchange between the A and P state. **b)** Similar to a) but here irradiation of the active state (A) leads to attenuation of the pre-active state (P) signal, again indicative of conformational exchange between the two states. The strongly overlapped signals of the A and P states required scaling of the saturation spectrum to enable a direct comparison of the signal intensities at the position of the peak of interest. The latter compensates for the loss in signal intensity at the peak of interest due to the reduced contribution from the attenuated saturated peak. Scaling factors were determined from the deconvolution shown in Supplementary Fig. 7b (right) whilst taking into account the residual intensity of the saturated peak in the saturated vs reference spectrum.

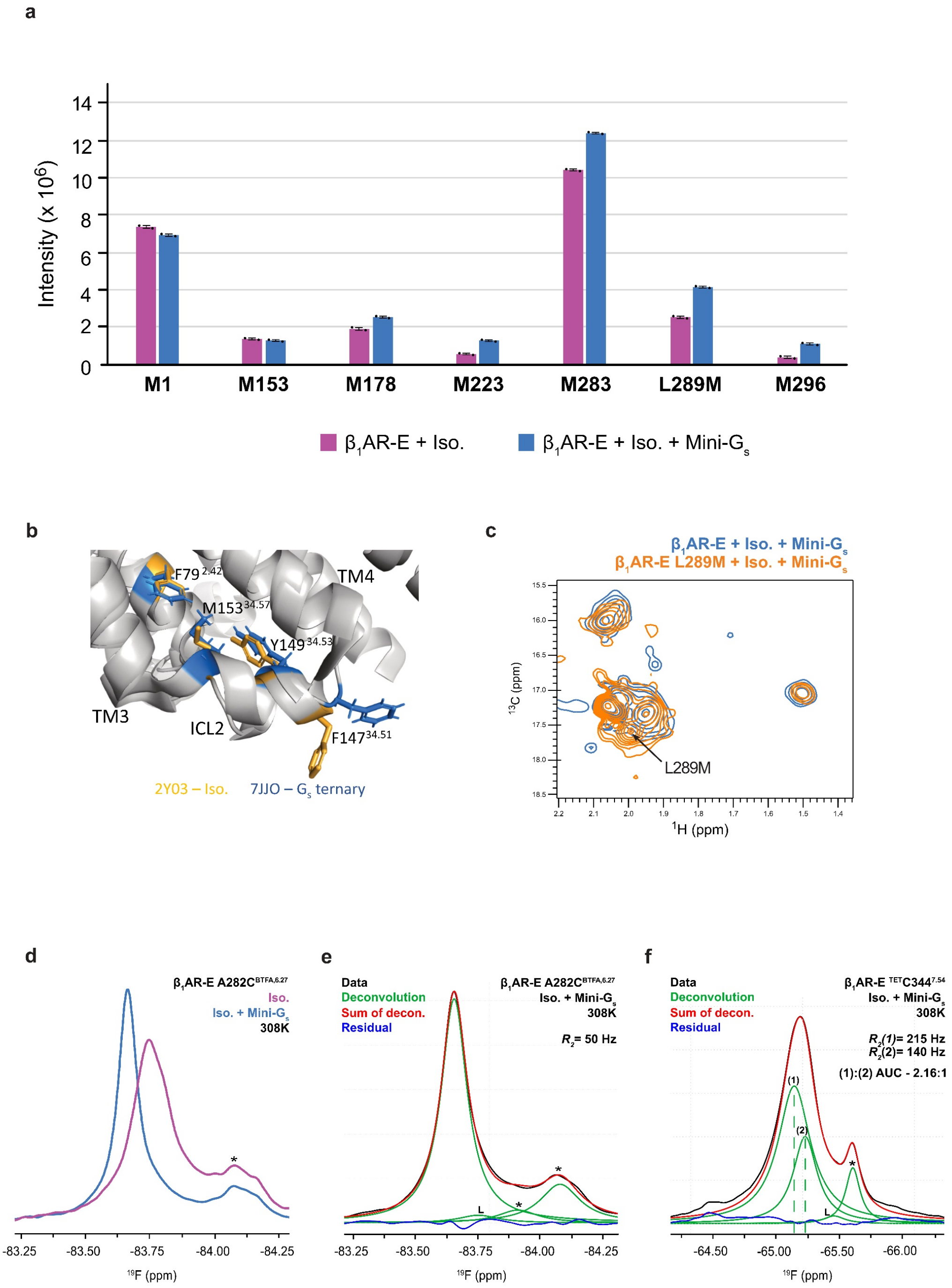

Supplementary Figure 9. β_1AR-E undergoes additional conformational changes upon ternary complex formation. **a)** A comparison of peak intensities from ^{1}H-^{13}C HMQC spectra of isoprenaline-bound (Iso.) β_1AR-E (purple) versus isoprenaline-bound β_1AR-E with 2 molar equivalents of mini-G$_s$ (blue). Note the largest relative difference between the two conditions are for M223$^{5.54}$ and M296$^{6.41}$. Error bars indicate SD between two repeats. **b)** A comparison of β_1AR structures showing the minor changes around the IL2 probe M153$^{34.57}$ between the isoprenaline-bound β_1AR crystal structure (orange - PDB: 2Y03)[12] and the isoprenaline-bound β_1AR-G$_s$ ternary cryo-EM structure (blue - PDB: 7JJO)[10]. **c)** An overlay of the ^{1}H-^{13}C HMQC NMR spectra of isoprenaline-bound (Iso.) β_1AR-E L289$^{6.34}$ with 2 molar equivalents of mini-G$_s$, and isoprenaline-bound β_1AR-E L289M$^{6.34}$ with 2 molar equivalents of mini-G$_s$, both recorded at 308 K. **d)** ^{19}F NMR spectra of β_1AR-E A282C$^{BTFA,6.27}$ in the isoprenaline-bound (Iso.) state (purple) and mini-G$_s$ (2 molar equivalents) ternary complex (blue), recorded at 308 K. * indicates a degradation product. **e)** Deconvolution of the β_1AR-E A282C$^{BTFA,6.27}$ ^{19}F NMR spectrum in the isoprenaline and mini-G$_s$ (2 molar equivalents) ternary complex, recorded at 308 K. Addition of mini-G$_s$

results in a single sharp ternary complex peak. The residual (blue) is the subtraction of the sum of the simulated peaks (Sum of decon., red) from the raw data (black). Individual simulated peaks are shown in green. * indicates known degradation products. L indicates residual ligand-bound population. R_2 transverse relaxation rate constant used to generate the simulated peak is indicated. **f)** Two-component deconvolution of the β_1AR-E ^{TET}C344$^{7.54}$ ^{19}F NMR spectrum in the isoprenaline and mini-G_s (2 molar equivalents) ternary complex, recorded at 308 K. The chemical shifts of two ternary complex states (1) and (2) are indicated with dashed green lines. Colours and symbols are as in **e)**. The deconvolution consisting of two components results in a fit with a residual error that is clearly below the noise in the spectrum. The ratio of the area under the curve (AUC) for the simulated (1) and (2) states is shown. The R_2 relaxation rate constants used to generate the simulated peaks are indicated.

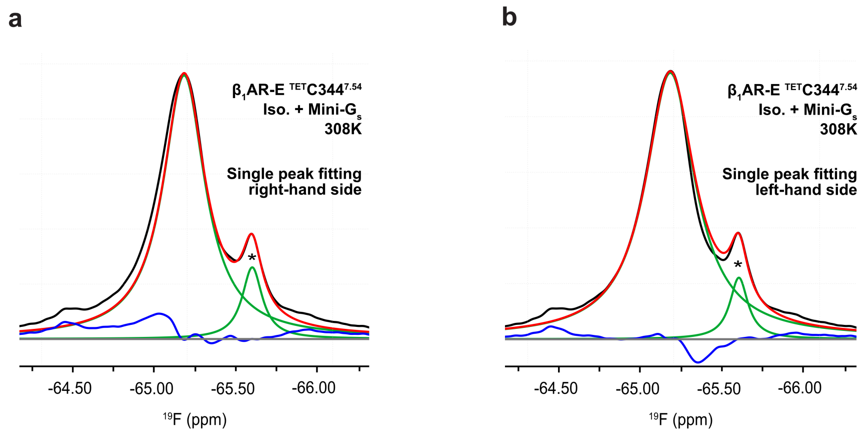

Supplementary Figure 10. Single-peak deconvolution of the TM7 ^{19}F NMR spectrum of β_1AR-E in ternary complex with isoprenaline and mini-G$_s$ indicate that the raw data for ^{TET}C344$^{7.54}$ cannot be fit by a single signal. a) Single-peak deconvolution of the spectrum shown in Supplementary Figure 9f (β_1AR-E ^{TET}C344$^{7.54}$ in the presence of isoprenaline and mini-G$_s$). The residual (blue) is the subtraction of the sum of the simulated peaks (Sum of decon., red) from the raw data (black). Individual simulated peaks are shown in green. * indicates known degradation products. The single major peak is fit to minimise the residual on the right-hand side of the peak, with the centre of the simulated peak matching the centre of the raw data. **b)** Alternative single-peak deconvolution with the single major peak fit to minimise the residual on the left-hand side of the peak, with the centre of the simulated peak matching the centre of the raw data. Details are as in **a)**. Both **a)** and **b)** are single major peak deconvolutions of the data shown in Supplementary Fig. 9f (two-component deconvolution) and produce fits with residual errors that are well above the noise in the spectrum.

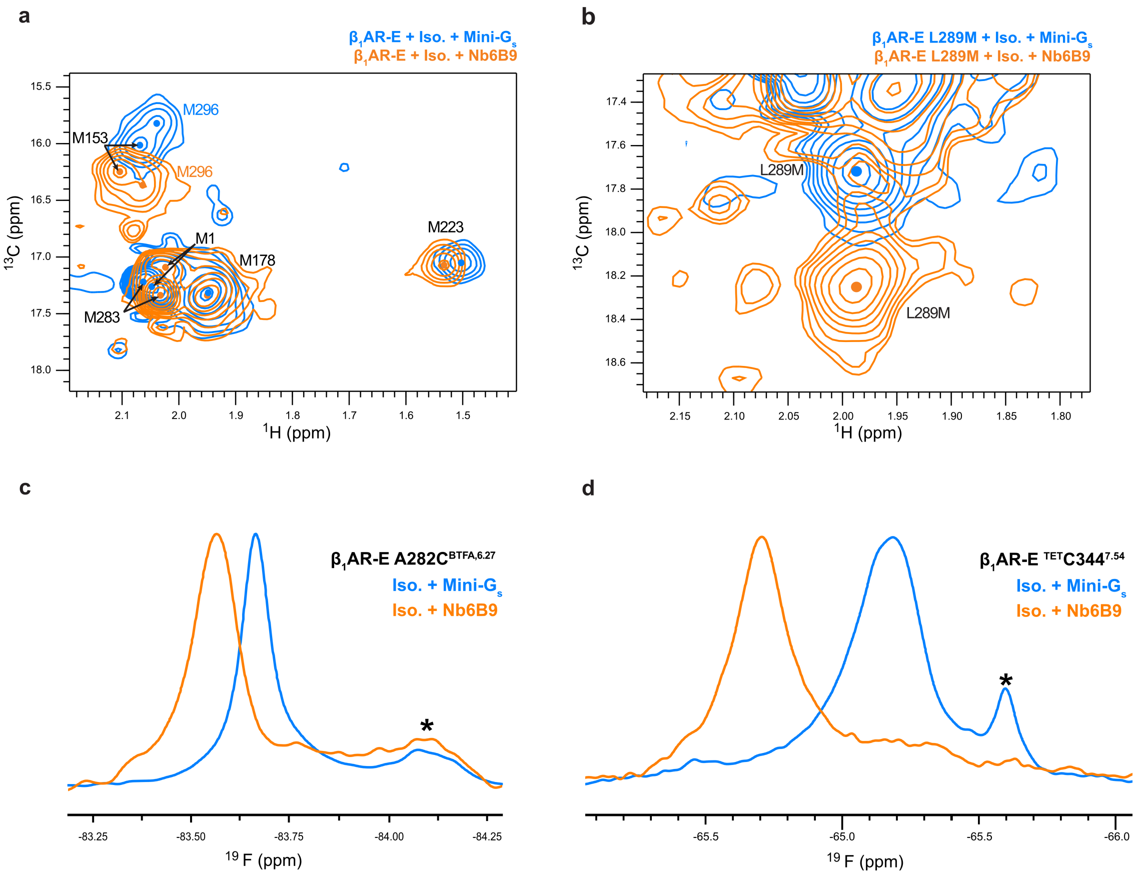

Supplementary Figure 11. Comparisons of isoprenaline-bound β$_1$AR-E ternary complex spectra with mini-G$_s$ or Nb6B9. a) An overlay of the ^{1}H-^{13}C HMQC NMR spectra of isoprenaline-bound (Iso.) β$_1$AR-E in complex with 2 molar equivalents mini-G$_s$ or Nb6B9 (used in our previous work[15,16]), recorded at 308 K. M178$^{4.62}$ showed negligible chemical shift differences and M223$^{5.54}$ small proton changes, suggesting similar arrangements around the orthosteric ligand pocket and conserved PIF region, respectively. M153$^{34.57}$ and M296$^{6.41}$ adopted downfield positions in the ^{13}C dimension for the mini-G$_s$ complex relative to the Nb6B9 complex, indicating trans χ^3 rotamers and thus reduced conformational exchange and a more rigid conformation for the mini-G$_s$ complex in IL2 and the core TM5-TM6 interface, respectively. The M283$^{6.28}$ proton shift in the Nb6B9 complex was shifted upfield relative to the mini-G$_s$ complex, further from the random coil value of 2.13 ppm[17] indicating the TM6 tip was less flexible and less solvent exposed. **b)** An overlay of the ^{1}H-^{13}C HMQC NMR spectra of isoprenaline-bound β$_1$AR-E L289M$^{6.34}$ in complex with 2 molar equivalents mini-G$_s$ or Nb6B9. Experimental conditions were identical to **a)** aside from the L289M$^{6.34}$ mutation. Similar proton shifts indicated a similar proximity of L289M$^{6.34}$ to Y231$^{5.62}$ thus similar rotations of TM6 between mini-G$_s$ and Nb6B9 ternary complexes. The downfield shift in ^{13}C for Nb6B9 indicated a χ^3 trans conformation and a lower degree of flexibility in TM6. **c)** ^{19}F NMR spectra of A282C$^{BTFA,6.27}$ in isoprenaline-bound β$_1$AR-E in complex with 2 molar equivalents mini-G$_s$ or Nb6B9, recorded at 308 K. The Nb6B9 complex is downfield shifted relative to mini-G$_s$ ternary complex and broader lines suggest differing conformational adjustments at the tip of TM6 and conformational broadening on a slower μs-to-ms timescale. **d)** ^{19}F NMR spectra of ^{TET}C344$^{7.54}$ in isoprenaline-bound β$_1$AR-E in complex with 2 molar equivalents mini-G$_s$ or Nb6B9, recorded at 308 K. The Nb6B9 complex broad peak was shifted downfield by 0.49 ppm relative to mini-G$_s$ likely due to differences in solvent accessibility, possibly indicating sub-optimal contacts between receptor and nanobody in the TM7/IL4 region relative to mini-G$_s$ interactions. Peaks are scaled to the same peak intensity in **c** and **d**.

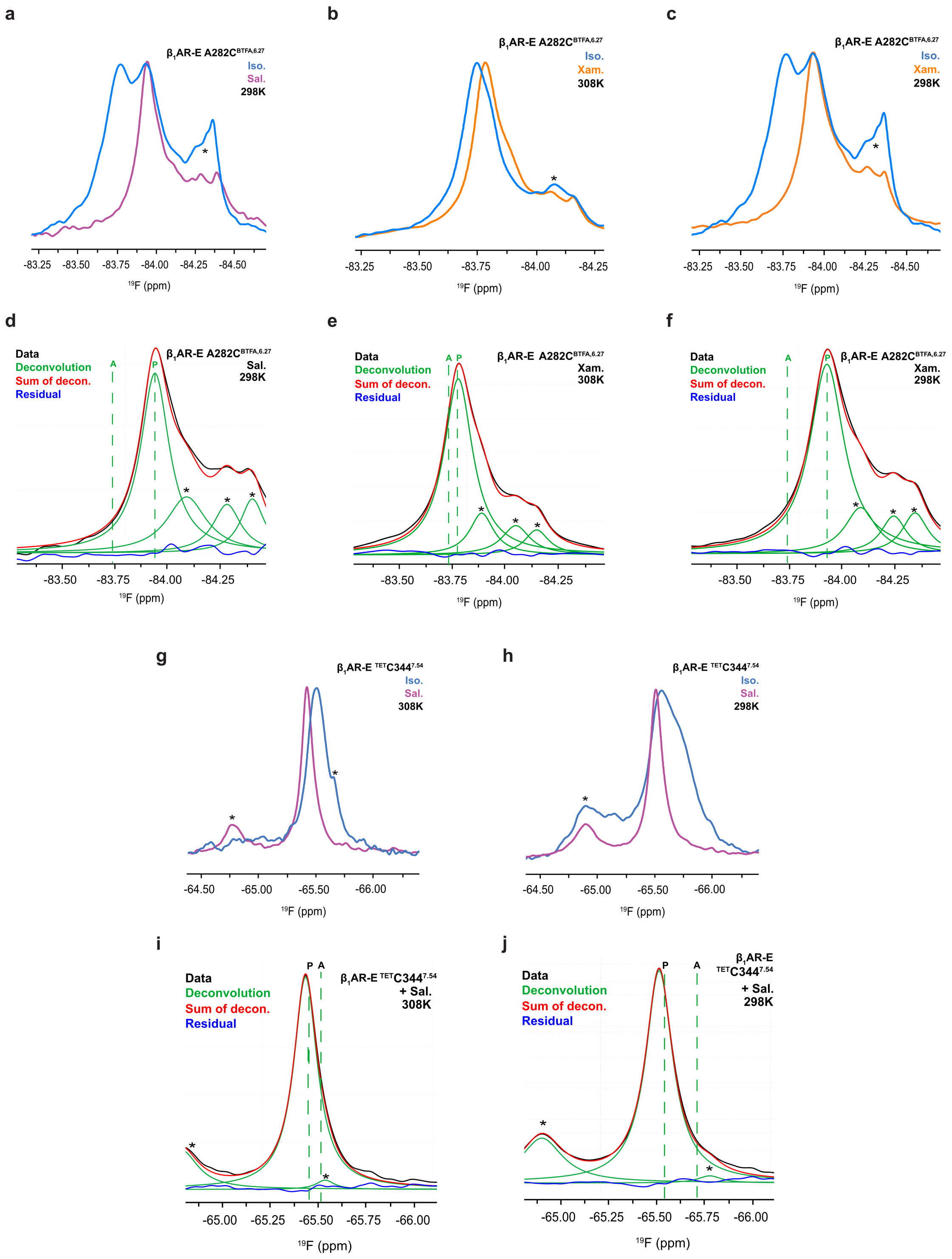

Supplementary Figure 12. ^{19}F NMR spectra show partial agonist bound β$_1$AR-E only populates the inactive/pre-active state equilibrium. **a)** ^{19}F NMR spectra of β$_1$AR-E A282C$^{BTFA,6.27}$ in the isoprenaline-bound (Iso.) state (blue) or salbutamol-bound (Sal.) state (purple), recorded at 298 K. * indicates degradation products. **b)** ^{19}F NMR spectra of β$_1$AR-E A282C$^{BTFA,6.27}$ in the isoprenaline-bound (Iso.) state (blue) or xamoterol-bound (Xam.) state (orange), recorded at 308 K. * indicates degradation products. **c)** ^{19}F NMR spectra of β$_1$AR-E A282C$^{BTFA,6.27}$ in the isoprenaline-bound (Iso.) state (blue) or xamoterol-bound (Xam.) state (orange), recorded at 298 K. * indicates degradation products. **d)** Deconvolution of the β$_1$AR-E A282C$^{BTFA,6.27}$ ^{19}F NMR spectrum in the presence of 1 mM salbutamol, recorded at 298 K. The green dashed lines indicate the chemical shifts of the active (A) and pre-active (P) states observed in deconvolutions of isoprenaline spectra.

The residual (blue) is the subtraction of the sum of the simulated peaks (Sum of decon., red) from the raw data (black). Individual simulated peaks are shown in green. * indicates degradation products. **e)** Deconvolution of the β_1AR-E A282C$^{BTFA,6.27}$ ^{19}F NMR spectrum in the presence of 1 mM xamoterol, recorded at 308 K. See **d)** for symbols and colours. **f)** Deconvolution of the β_1AR-E A282C$^{BTFA,6.27}$ ^{19}F NMR spectrum in the presence of 1 mM xamoterol, recorded at 298 K. See **d)** for symbols and colours. **g)** ^{19}F NMR spectra of β_1AR-E ^{TET}C344$^{7.54}$ in the isoprenaline-bound state (blue) or salbutamol-bound state (purple), recorded at 308 K. * indicates degradation products. **h)** ^{19}F NMR spectra of β_1AR-E ^{TET}C344$^{7.54}$ in the isoprenaline-bound state (blue) or salbutamol-bound state (purple), recorded at 298 K. * indicates degradation products. **i)** Deconvolution of the β_1AR-E ^{TET}C344$^{7.54}$ ^{19}F NMR spectrum in the presence of 1 mM salbutamol, recorded at 308 K. See **d)** for symbols and colours. **j)** Deconvolution of the β_1AR-E ^{TET}C344$^{7.54}$ ^{19}F NMR spectrum in the presence of 1 mM salbutamol, recorded at 298 K. See **d)** for symbols and colours. Note for **a-c**, and **g-h**, peaks were scaled to the same intensity maxima.

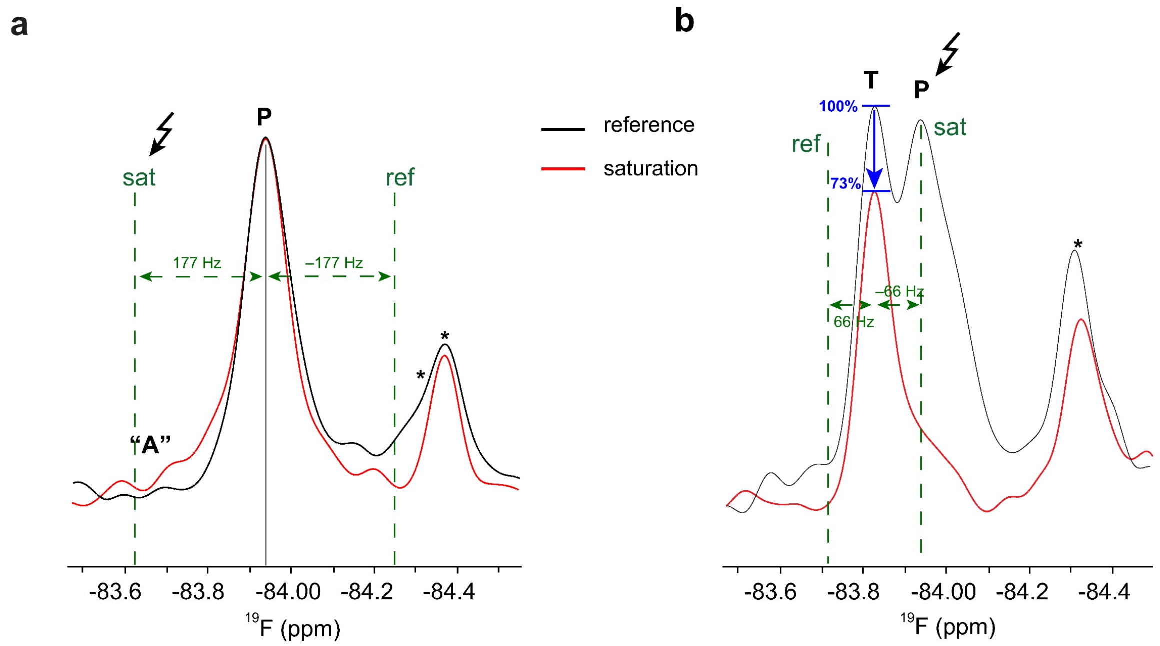

Supplementary Figure 13. ^{19}F NMR saturation transfer experiments on β$_1$AR-E A282C$^{BTFA,6.27}$ indicate partial agonists induce ternary complex formation via the pre-active state for β$_1$AR. Pairwise 1D ^{19}F NMR experiments were recorded following irradiation with a weak RF field (irradiation time of 1 s, saturation field strength 25 Hz) applied at either of the two offset positions located symmetrically (dashed green lines with the corresponding offset positions of the saturation field indicated) around the peak of interest, which resulted in a saturation spectrum (red, irradiation at position 'sat') or a reference spectrum (black, irradiation at position 'ref'), respectively. At the corresponding position of the peak of interest, the reduction in signal intensity due to saturation transfer is indicated by a blue arrow, with the signal intensity in the saturation spectrum given as a percentage of the intensity in the reference spectrum. **a)** β$_1$AR-E A282C$^{BTFA,6.27}$ bound to xamoterol (500 μM) recorded at 298 K. Saturation of the active state position (using the same offset from the pre-active state signal as in Supplementary Fig. 8, right, 177 Hz) had a negligible effect on the pre-active state signal, indicating partial agonist-bound β$_1$AR-E does not occupy the active state under these conditions. **b)** β$_1$AR-E A282C$^{BTFA,6.27}$ in ternary complex with xamoterol (500 μM) and mini-G$_s$ (0.75 molar equivalents). Experiments were recorded at 308 K to increase the separation of signals. Saturation of the pre-active state signal resulted in a decrease in the ternary complex signal, indicating chemical exchange between the pre-active state (P) and ternary complex (T). The saturation spectrum is shown scaled relative to its reference spectrum, in order to correct for the intensity loss at the signal position of the ternary complex (T) due to overlap with the saturated pre-active state (P) peak (see Supplementary Fig. 8).

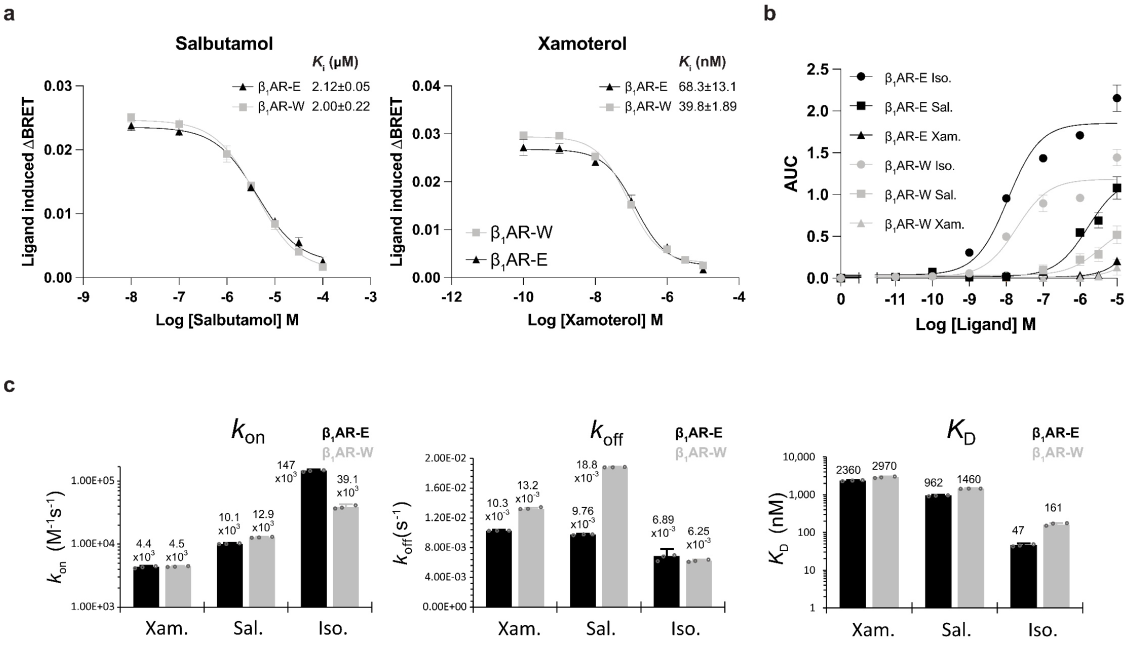

Supplementary Figure 14. Partial agonists reduce G protein coupling and mini-G_s binding affinities to β_1AR constructs relative to full agonist. a) Ligand binding affinities of the β_1AR constructs for salbutamol (left) and xamoterol (right) expressed in HEK293T cells, measured using the luminescence-based ligand binding assay shown in Supplementary Fig. 2a. K_i values are shown in inset. **b)** *In cell* measurements of the dissociation of the G protein Gβγ subunit from $G\alpha_s$ using the TRUPATH assay system, shown in Supplementary Fig. 2c, for partial agonists salbutamol (Sal., squares) and xamoterol (Xam., triangles) relative to full agonist isoprenaline (Iso., circles). The responses were measured for varying ligand concentrations to obtain EC_{50} curves for β_1AR-E (black) and β_1AR-W (grey). Data are mean ± SEM of n=3 independent biological experiments, each performed in duplicate. **c)** BLI kinetic data for binding of β_1AR to mini-G_s in the presence of different ligands, xamoterol (Xam.), salbutamol (Sal.) and isoprenaline (Iso.). Black, β_1AR-E, grey, β_1AR-W. Values were the average of triplicates (n=3 individual repeats), and error bars indicate SD of these triplicates. Note for the logarithmic axes for k_{on} and K_D data. See Methods and Supplementary Methods for details.

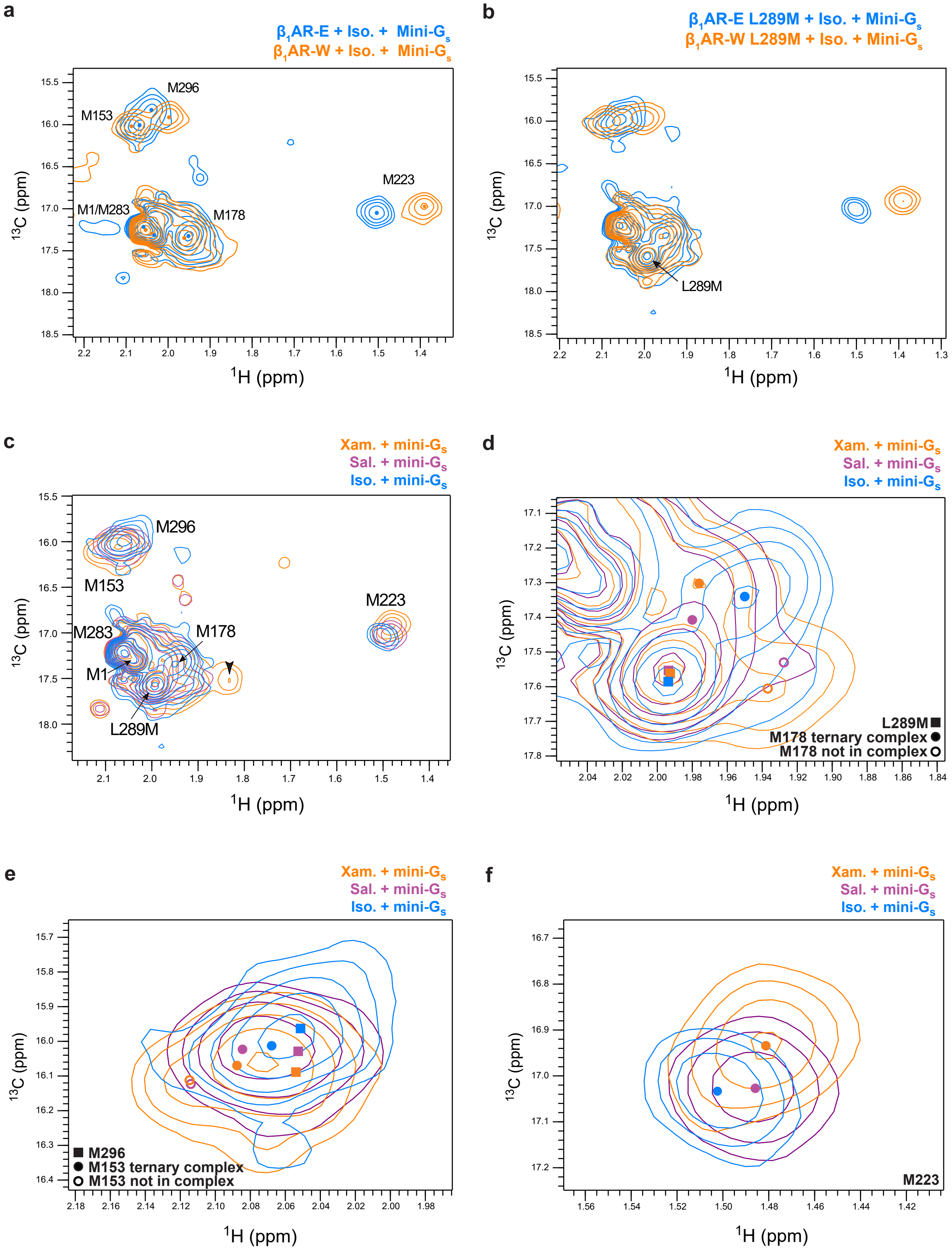

Supplementary Figure 15. ^{1}H-^{13}C HMQC NMR spectra show E130W$^{3.41}$ thermostabilising mutation and partial agonists affect ternary complex conformations. a) An overlay of the ^{1}H-^{13}C HMQC NMR spectra of isoprenaline-bound (Iso.) β_1AR-E with 2 molar equivalents of mini-G_s (blue) and isoprenaline-bound β_1AR-W with 2 molar equivalents of mini-

G_s (orange), both recorded at 308 K. **b)** An overlay of the ^{1}H-^{13}C HMQC NMR spectra of isoprenaline-bound β_1AR-E L289M$^{6.34}$ with 2 molar equivalents of mini-G_s (blue) and isoprenaline-bound β_1AR-W L289M$^{6.34}$ with 2 molar equivalents of mini-G_s (orange), both recorded at 308 K. **c)** An overlay of the ^{1}H-^{13}C HMQC NMR spectra of mini-G_s bound β_1AR-E L289M$^{6.34}$ recorded at 308 K in the presence of the ligands xamoterol (Xam., orange), salbutamol (Sal., purple), or isoprenaline (Iso., blue). The arrowhead indicates a residual ligand-bound population. **d)** Enlargement of **c)** in the M178$^{4.62}$/L289M$^{6.34}$ region. M178$^{4.62}$ peak positions are shown with filled circles and L289M$^{6.34}$ peak positions are shown with squares. Residual M178$^{4.62}$ ligand-bound peaks are shown with unfilled circles. **e)** Enlargement of **c)** in the M153$^{34.57}$/M296$^{6.41}$ region. M153$^{34.57}$ peak positions are shown with filled circles and M296$^{6.41}$ peak positions are shown with squares. Residual M153$^{34.57}$ ligand-bound peaks are shown with unfilled circles. **f)** Enlargement of **c)** in the M223$^{5.54}$ region.

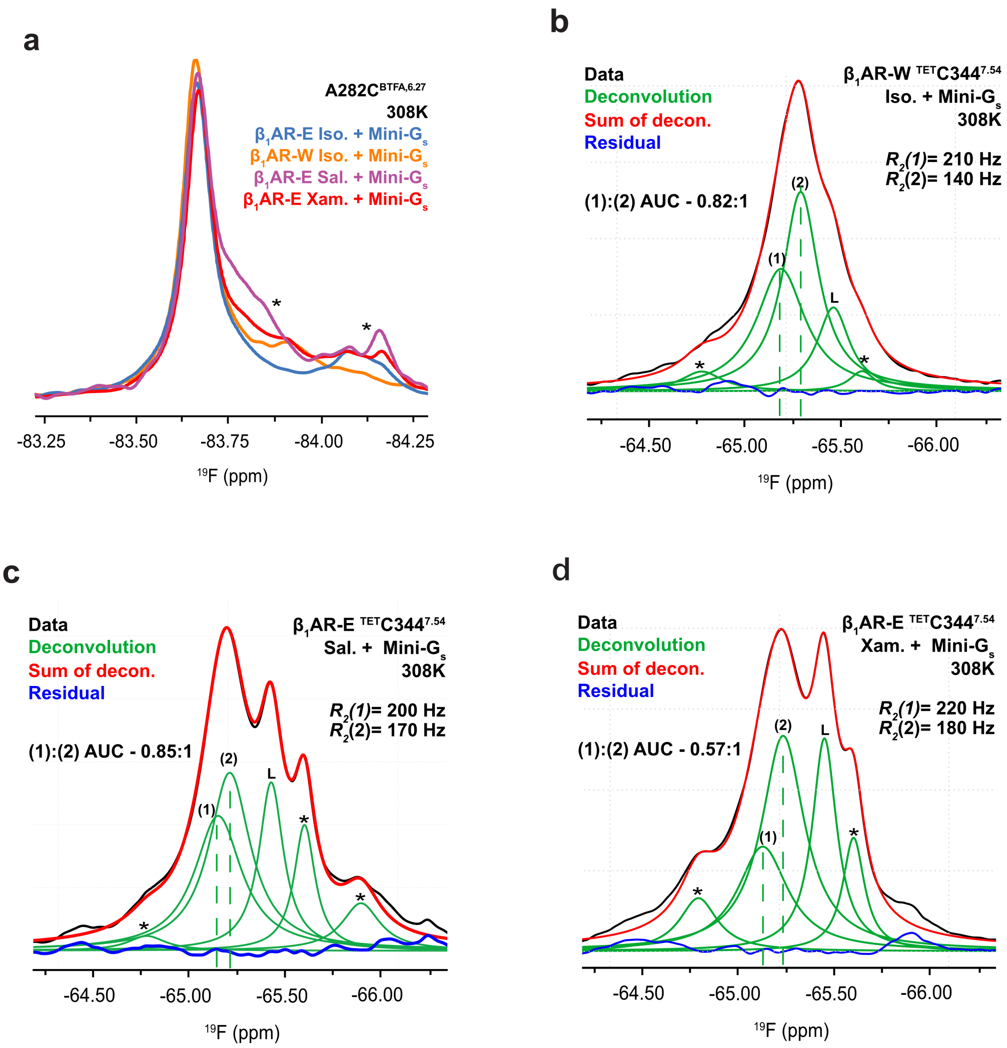

Supplementary Figure 16. ^{19}F NMR spectra show E130W$^{3.41}$ thermostabilising mutation and partial agonists affect ternary complex conformations. **a)** ^{19}F NMR spectra of A282C$^{BTFA,6.27}$ in the isoprenaline (Iso.) and mini-G$_s$ ternary (2 molar equivalents) complex for β$_1$AR-E (blue) or β$_1$AR-W (orange), and A282C$^{BTFA,6.27}$ in the salbutamol (Sal., high efficacy partial agonist, purple) or xamoterol (Xam., intermediate efficacy partial agonist, red) ternary complexes with mini-G$_s$ (8 molar equivalents) for β$_1$AR-E, recorded at 308 K. * indicates degradation products. **b)** Deconvolution of the β$_1$AR-W ^{TET}C344$^{7.54}$ ^{19}F NMR spectrum in the isoprenaline and mini-G$_s$ (2 equivalents) ternary complex, recorded at 308 K. The chemical shifts of two ternary complex states (1) and (2) are indicated with dashed green lines. The residual (blue) is the subtraction of the sum of the simulated peaks (Sum of decon., red) from the raw data (black). Individual simulated peaks are shown in green. * indicates known degradation products. L indicates residual ligand-bound population. The ratio of the area under the curve (AUC) for the simulated (1) and (2) states is shown. The R_2 transverse relaxation rate constants used to generate the simulated peaks are indicated. **c)** Deconvolution of the β$_1$AR-E ^{TET}C344$^{7.54}$ ^{19}F NMR spectrum in the salbutamol and mini-G$_s$ (8 molar equivalents) ternary complex, recorded at 308 K. Symbols (1), (2), *, L, AUC, and R_2 are as in **b)**. See **b)** for line colours. **d)** Deconvolution of the β$_1$AR-E ^{TET}C344$^{7.54}$ ^{19}F NMR spectrum in the xamoterol and mini-G$_s$ (8 molar equivalents) ternary complex, recorded at 308 K. Symbols (1), (2), *, L, AUC, and R_2 are as in **b)**. See **b)** for line colours.

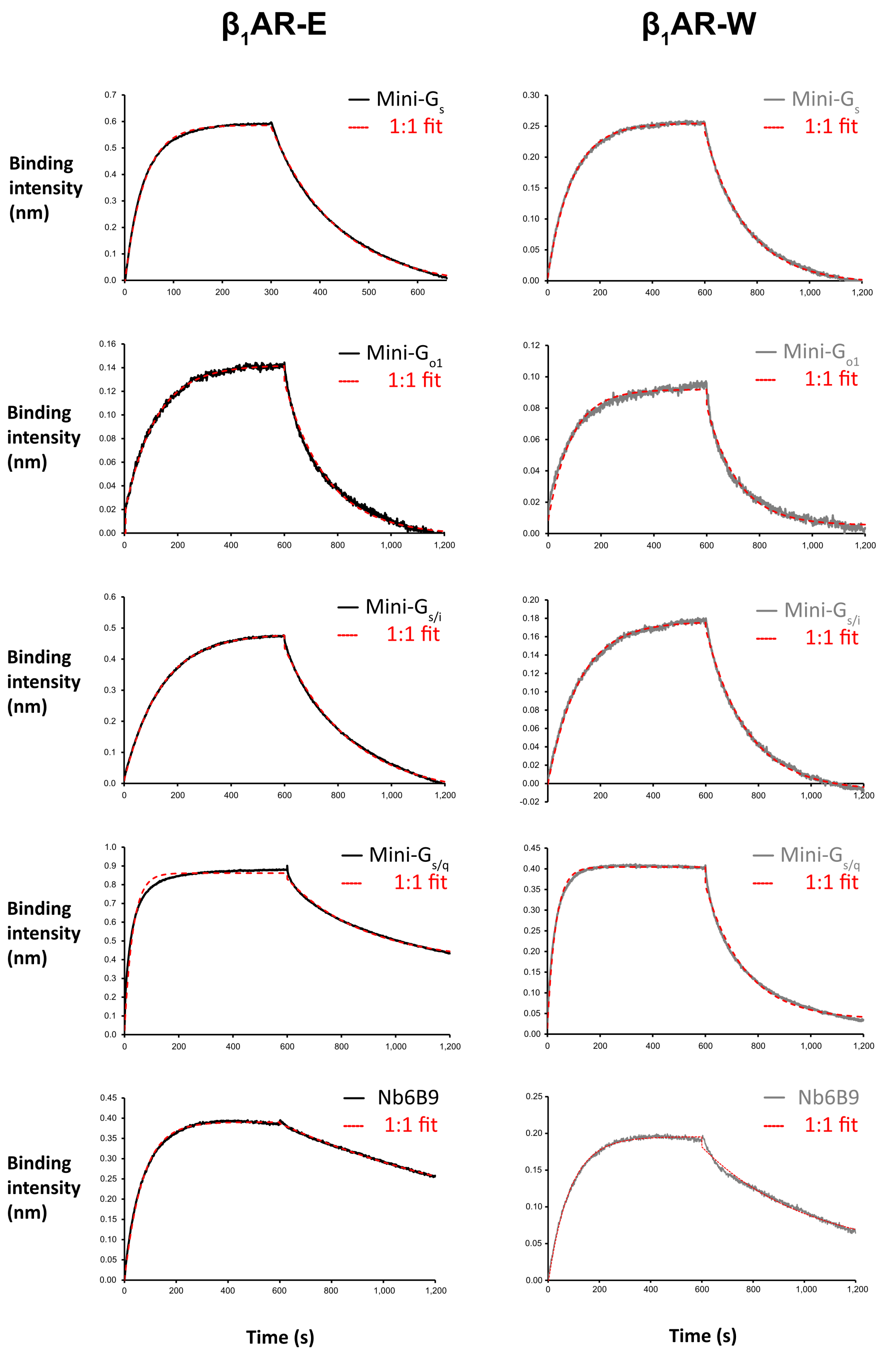

Supplementary Figure 17. Examples of isotherms for BLI experiments assessing β$_1$AR binding kinetics to mini-G proteins and Nb6B9. Left, β$_1$AR-E, right, β$_1$AR-W. All binding curves were recorded in the presence of 500 µM isoprenaline. Raw BLI data is shown as solid black lines for β$_1$AR-E and solid grey lines for β$_1$AR-W, and 1:1 stoichiometry model fits are shown as red dashed lines. Isotherms are representative data of n=3 individual replicates. See Methods and Supplementary Methods for experimental details. See Supplementary Table 4. for the concentrations of analyte used.

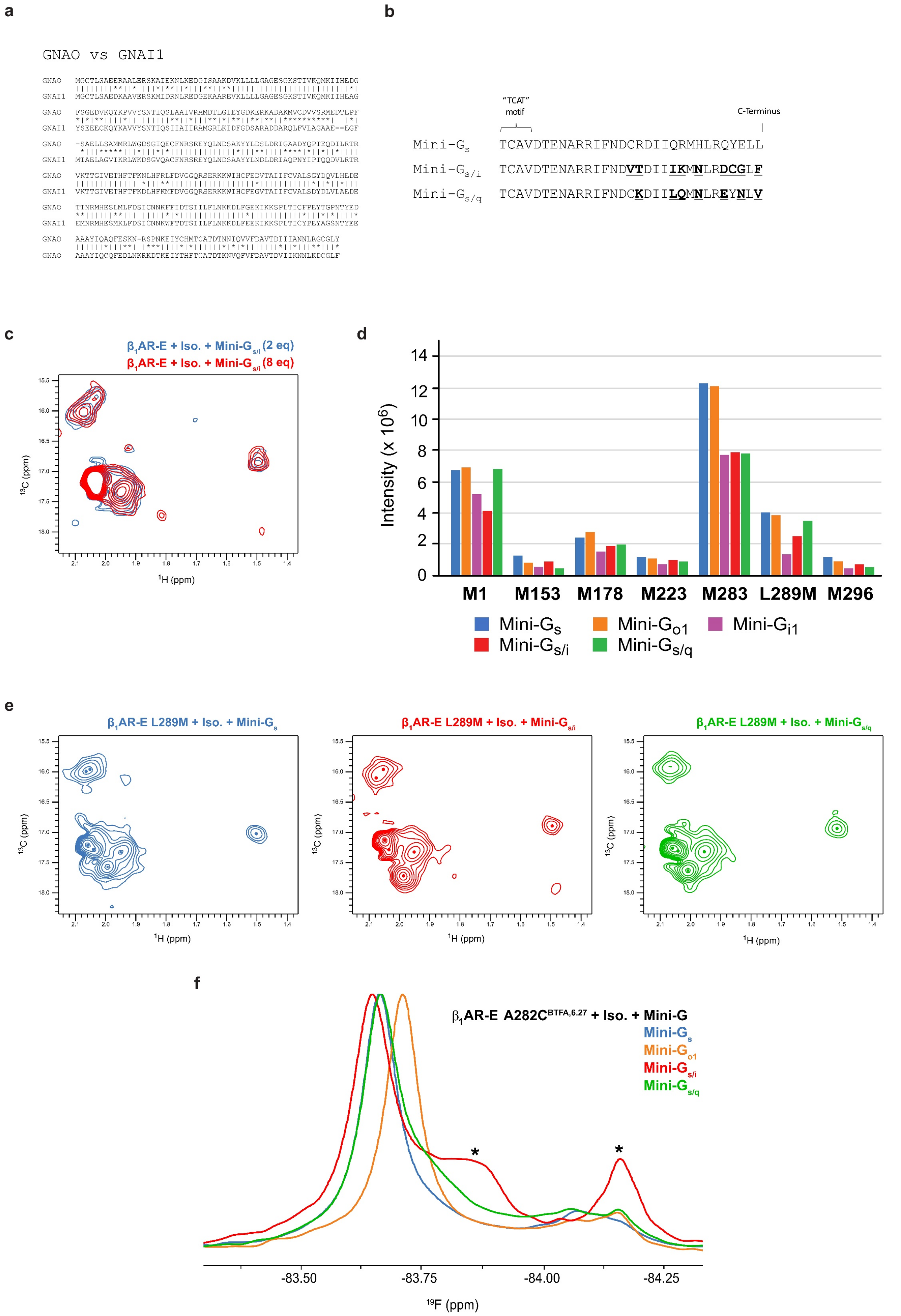

Supplementary Figure 18. Differences in β$_1$AR-E conformation in ternary complexes with non-chimeric and chimeric mini-G proteins. a) A sequence alignment of the GNAO (G_{o1}) sequence (Uniprot accession code: P09471) and the GNAI1 (G_{i1}) sequence (Uniprot accession code: P63096). Identity is indicated with |, differences are indicated with *,

gaps are indicated with -. **b)** Sequence alignments of the mini-G_s protein with the mini-G chimeras mini-$G_{s/i}$ and mini-$G_{s/q}$ from the TCAT motif to the C-terminus[18]. Differences between the chimeras and the mini-G_s protein are shown underlined and in bold. **c)** Representative ^{1}H-^{13}C HMQC NMR spectra of isoprenaline-bound β_1AR-E ternary complexes with 2 or 8 equivalents of mini-G proteins. Shown is an example for mini-$G_{s/i}$. The minimal differences between spectra indicate negligible binding kinetics-induced changes. **d)** A comparison of peak intensities from ^{1}H-^{13}C HMQC spectra of isoprenaline-bound (Iso.) β_1AR-E in complex with 2 molar equivalents of different mini-G binding partners. **e)** An overlay of the ^{1}H-^{13}C HMQC NMR spectra of isoprenaline-bound (Iso.) β_1AR-E L289M$^{6.34}$ in complex with different mini-G chimeras recorded at 308 K. Left, mini-G_s, middle, mini-$G_{s/i}$, right, mini-$G_{s/q}$. All experiments used 2 molar equivalents of mini-G protein. **f)** An overlay of the ^{19}F NMR spectra of isoprenaline-bound β_1AR-E A282C$^{BTFA,6.27}$ in complex with different mini-G proteins recorded at 308 K. The spectrum with mini-G_s used 2 molar equivalents of mini-G protein, all other spectra used 8 molar equivalents.

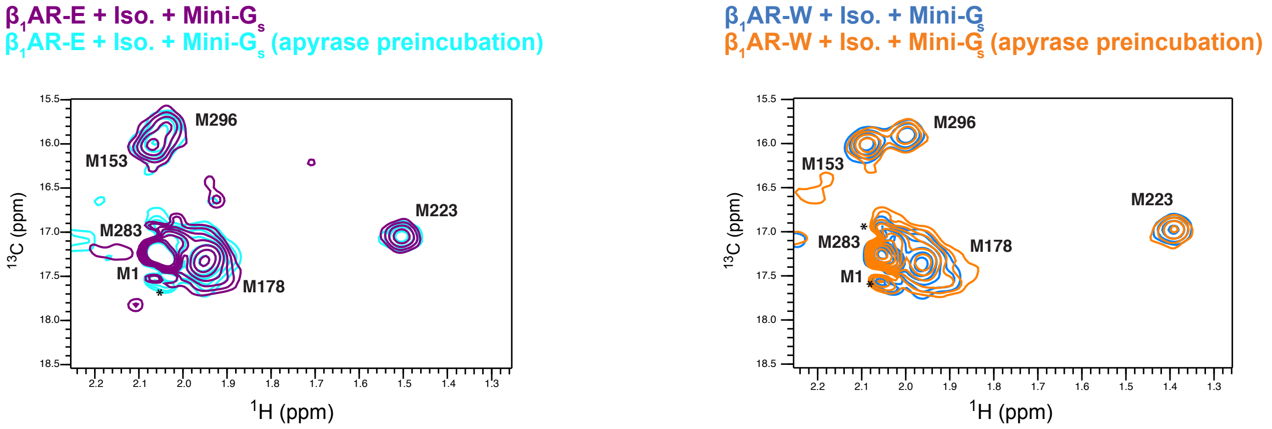

Supplementary Figure 19. Ternary complex spectra appearance does not depend on the presence of GDP. Superposition of 2D ^{1}H-^{13}C HMQC correlation spectra of β_1AR-E (left) and β_1AR-W (right) in ternary complex bound to isoprenaline and mini-G_s. The spectra were recorded either following preincubation with apyrase (5 units) for two hours (cyan for β_1AR-E, orange for β_1AR-W) or directly without preincubation (purple for β_1AR-E, blue for β_1AR-W). The signals of the ternary complexes are independent of the presence of GDP in solution, in agreement with our BLI experiments (Supplementary Fig. 2f) and consistent with a mutation in the mini-G proteins that makes them insensitive to GDP nucleotide binding when coupled to GPCRs[5]. Based on this comparison, all NMR and BLI experiments in this work were recorded without prior apyrase incubation. * indicates an artifact related to ^{1}H pulse imperfections.

Supplementary Table 1. Comparison of BLI binding kinetics for β_1AR association with mini-G_s in the presence of 500 µM isoprenaline using different modelling parameters. Single point measurements (single optimised concentrations), dose-response measurements (with global fitting), and steady state response measurements (using response curves following the equation Response = (R_{max} * $Concentration_{analyte}$)/(K_D + $Concentration_{analyte}$)). Values shown are triplicate averages (n=3 individual repeats) with ± one standard deviation.

Measurement method	Receptor Construct	K_D (M)	k_{on} ($M^{-1}s^{-1}$)	k_{off} (s^{-1})
Single point	β_1AR-E	$4.68x10^{-8}$ $\pm 4.18x10^{-9}$	$1.47x10^{5}$ $\pm 6.86x10^{3}$	$6.89x10^{-3}$ $\pm 9.10x10^{-4}$
Dose-response	β_1AR-E	$5.81x10^{-8}$ $\pm 1.79x10^{-8}$	$1.21x10^{5}$ $\pm 1.37x10^{4}$	$6.89x10^{-3}$ $\pm 1.53x10^{-3}$
Steady state	β_1AR-E	$4.30x10^{-8}$ $\pm 3.90x10^{-9}$	N/A	N/A
Single point	β_1AR-W	$1.61x10^{-7}$ $\pm 1.79x10^{-8}$	$3.91x10^{4}$ $\pm 4.04x10^{3}$	$6.25x10^{-3}$ $\pm 1.37x10^{-4}$
Dose-response	β_1AR-W	$1.81x10^{-7}$ $\pm 1.98x10^{-8}$	$5.18x10^{4}$ $\pm 1.54x10^{3}$	$9.40x10^{-3}$ $\pm 7.78x10^{-4}$
Steady state	β_1AR-W	$1.80x10^{-7}$ $\pm 3.20x10^{-8}$	N/A	N/A

Supplementary Table 2. BLI kinetic data for Nb6B9 binding to β_1AR constructs. Kinetic values for binding of β_1AR-E or β_1AR-W to active state stabilising nanobody Nb6B9 in the presence of 500 µM full agonist isoprenaline. Values shown are triplicate averages (n=3 individual repeats) with ± one standard deviation. See Methods and Supplementary Methods for details.

	β_1AR-E		β_1AR-W	
k_{on} ($M^{-1} s^{-1}$)	1.05×10^{5}	$\pm 1.44 \times 10^{3}$	7.74×10^{4}	$\pm 5.32 \times 10^{3}$
k_{off} (s^{-1})	8.93×10^{-4}	$\pm 1.13 \times 10^{-4}$	2.49×10^{-3}	$\pm 1.62 \times 10^{-4}$
K_D (M)	8.53×10^{-9}	$\pm 9.60 \times 10^{-10}$	3.23×10^{-8}	$\pm 3.99 \times 10^{-9}$

Supplementary Table 3. BLI kinetic data for mini-G_s binding to β_1AR constructs in the presence of different agonists. Kinetic values for mini-G_s binding to β_1AR-E or β_1AR-W in the presence of 500 μM of different ligands (isoprenaline – Iso., full agonist; salbutamol – Sal., partial agonist (high efficacy); xamoterol – Xam., partial agonist (intermediate efficacy))[19]. Top, k_{on} values, middle, k_{off} values, bottom, K_D values. Values shown are triplicate averages (n=3 individual repeats) with ± one standard deviation. See Methods and Supplementary Methods for details.

k_{on} ($M^{-1}s^{-1}$)

	β_1AR-E	β_1AR-W
Iso.	$1.47x10^{5}$ $\pm6.86x10^{3}$	$3.91x10^{4}$ $\pm4.04x10^{3}$
Sal.	$1.01x10^{4}$ $\pm1.83x10^{2}$	$1.29x10^{4}$ $\pm1.93x10^{2}$
Xam.	$4.36x10^{3}$ $\pm1.12x10^{2}$	$4.46x10^{3}$ $\pm1.55x10^{2}$

k_{off} (s^{-1})

	β_1AR-E	β_1AR-W
Iso.	$6.89x10^{-3}$ $\pm9.10x10^{-4}$	$6.25x10^{-3}$ $\pm1.37x10^{-4}$
Sal.	$9.76x10^{-3}$ $\pm5.99x10^{-5}$	$1.88x10^{-2}$ $\pm6.96x10^{-5}$
Xam.	$1.03x10^{-2}$ $\pm8.46x10^{-5}$	$1.32x10^{-2}$ $\pm1.40x10^{-4}$

K_D (M)

	β_1AR-E	β_1AR-W
Iso.	$4.68x10^{-8}$ $\pm4.18x10^{-9}$	$1.61x10^{-7}$ $\pm1.79x10^{-8}$
Sal.	$9.62x10^{-7}$ $\pm1.59x10^{-8}$	$1.46x10^{-6}$ $\pm1.66x10^{-8}$
Xam.	$2.36x10^{-6}$ $\pm4.38x10^{-8}$	$2.97x10^{-6}$ $\pm8.30x10^{-8}$

Supplementary Table 4. BLI binding partner concentrations used for k_{on}, k_{off} and K_D determination based on single-concentration measurements. The binding partner concentrations correspond to conditions as established by pre-inspection of a concentration series that identified a BLI trace with a clear 1:1 monophasic behaviour (see Supplementary Methods).

Receptor Construct	β_1AR ligand (500 µM)	Binding partner	Binding partner concentration (nM)
β_1AR-E	Isoprenaline	Mini-G_s	62.5
β_1AR-E	Isoprenaline	Mini-G_{o1}	2000
β_1AR-E	Isoprenaline	Mini-$G_{s/i}$	1000
β_1AR-E	Isoprenaline	Mini-$G_{s/q}$	2000
β_1AR-E	Salbutamol	Mini-G_s	2000
β_1AR-E	Xamoterol	Mini-G_s	4000
β_1AR-E	Isoprenaline	Nb6B9	125
β_1AR-W	Isoprenaline	Mini-G_s	125
β_1AR-W	Isoprenaline	Mini-G_{o1}	4000
β_1AR-W	Isoprenaline	Mini-$G_{s/i}$	1000
β_1AR-W	Isoprenaline	Mini-$G_{s/q}$	2000
β_1AR-W	Salbutamol	Mini-G_s	1000
β_1AR-W	Xamoterol	Mini-G_s	4000
β_1AR-W	Isoprenaline	Nb6B9	125

Supplementary Table 5. BLI kinetic data for binding of mini-G proteins to β_1AR constructs. Kinetic values for binding of β_1AR-E or β_1AR-W to different mini-G proteins in the presence of 500 μM full agonist isoprenaline. Top, k_{on} values, middle, k_{off} values, bottom, K_D values. Values shown are triplicate averages (n=3 individual repeats) with ± one standard deviation. See Methods and Supplementary Methods for details.

k_{on} ($M^{-1}s^{-1}$)

	β_1AR-E	β_1AR-W
Mini-G_s	1.47×10^5 $\pm 6.86 \times 10^3$	3.91×10^4 $\pm 4.04 \times 10^3$
Mini-G_{o1}	2.96×10^3 $\pm 2.31 \times 10^2$	1.32×10^3 $\pm 3.22 \times 10^2$
Mini-$G_{s/i}$	3.64×10^3 $\pm 2.45 \times 10^2$	2.51×10^3 $\pm 4.42 \times 10^2$
Mini-$G_{s/q}$	1.41×10^4 $\pm 1.58 \times 10^2$	1.25×10^4 $\pm 1.84 \times 10^2$

k_{off} (s^{-1})

	β_1AR-E	β_1AR-W
Mini-G_s	6.89×10^{-3} $\pm 9.10 \times 10^{-4}$	6.25×10^{-3} $\pm 1.37 \times 10^{-4}$
Mini-G_{o1}	3.93×10^{-3} $\pm 3.17 \times 10^{-4}$	9.41×10^{-3} $\pm 6.54 \times 10^{-4}$
Mini-$G_{s/i}$	3.97×10^{-3} $\pm 3.97 \times 10^{-5}$	6.23×10^{-3} $\pm 1.55 \times 10^{-4}$
Mini-$G_{s/q}$	3.43×10^{-3} $\pm 2.23 \times 10^{-5}$	6.70×10^{-3} $\pm 1.06 \times 10^{-4}$

K_D (M)

	β_1AR-E	β_1AR-W
Mini-G_s	4.68×10^{-8} $\pm 4.18 \times 10^{-9}$	1.61×10^{-7} $\pm 1.79 \times 10^{-8}$
Mini-G_{o1}	1.34×10^{-6} $\pm 1.88 \times 10^{-7}$	7.41×10^{-6} $\pm 1.75 \times 10^{-6}$
Mini-$G_{s/i}$	1.09×10^{-6} $\pm 7.82 \times 10^{-8}$	2.53×10^{-6} $\pm 4.64 \times 10^{-7}$
Mini-$G_{s/q}$	2.42×10^{-7} $\pm 1.19 \times 10^{-9}$	5.34×10^{-7} $\pm 6.22 \times 10^{-9}$

Supplementary Table 6. Exact p values for comparison of binding kinetics parameters between mini-G proteins for BLI experiments with β_1AR-E in Figure 4c. The p values were calculated using unpaired two-tailed t-tests using the means and standard deviations in Supplementary Table 5, with a Bonferroni correction to the significance level for multiple comparisons. Significance level: p = 0.05, corrected to p = 0.00833.

Parameter	Comparison 1	Comparison 2	p value	Significant
k_{on}	Mini-G_s	Mini-$G_{s/i}$	0.0007535	Yes
k_{on}	Mini-G_s	Mini-G_{o1}	0.0007476	Yes
k_{on}	Mini-G_s	Mini-$G_{s/q}$	0.0008846	Yes
k_{on}	Mini-$G_{s/i}$	Mini-G_{o1}	0.02603	No
k_{on}	Mini-$G_{s/i}$	Mini-$G_{s/q}$	2.44e-06	Yes
k_{on}	Mini-G_{o1}	Mini-$G_{s/q}$	1.168e-06	Yes
k_{off}	Mini-G_s	Mini-$G_{s/i}$	0.03059	No
k_{off}	Mini-G_s	Mini-G_{o1}	0.02074	No
k_{off}	Mini-G_s	Mini-$G_{s/q}$	0.02221	No
k_{off}	Mini-$G_{s/i}$	Mini-G_{o1}	0.8716	No
k_{off}	Mini-$G_{s/i}$	Mini-$G_{s/q}$	0.0001875	Yes
k_{off}	Mini-G_{o1}	Mini-$G_{s/q}$	0.1098	No
K_D	Mini-G_s	Mini-$G_{s/i}$	0.001808	Yes
K_D	Mini-G_s	Mini-G_{o1}	0.007003	Yes
K_D	Mini-G_s	Mini-$G_{s/q}$	5.01e-05	Yes
K_D	Mini-$G_{s/i}$	Mini-G_{o1}	0.1431	No
K_D	Mini-$G_{s/i}$	Mini-$G_{s/q}$	0.002796	Yes
K_D	Mini-G_{o1}	Mini-$G_{s/q}$	0.009721	No

Supplementary Table 7. Exact p values for comparisons of binding kinetics parameters between β_1AR-E and β_1AR-W for each mini-G protein in BLI experiments in Figure 4c. The p values were calculated using unpaired two-tailed t-tests using the means and standard deviations in Supplementary Table 5. Significance level: p = 0.05.

Parameter	Mini-G protein	p value	Significant
k_{on}	Mini-G_s	9.992e-05	Yes
k_{on}	Mini-G_{o1}	0.002878	Yes
k_{on}	Mini-$G_{s/i}$	0.02884	Yes
k_{on}	Mini-$G_{s/q}$	0.0003839	Yes
k_{off}	Mini-G_s	0.3504	No
k_{off}	Mini-G_{o1}	0.001374	Yes
k_{off}	Mini-$G_{s/i}$	0.000861	Yes
k_{off}	Mini-$G_{s/q}$	0.0002065	Yes
K_D	Mini-G_s	0.005893	Yes
K_D	Mini-G_{o1}	0.02549	Yes
K_D	Mini-$G_{s/i}$	0.03005	Yes
K_D	Mini-$G_{s/q}$	9.11e-05	Yes

Supplementary Table 8. List of primers used in this study.

Primer Name	Primer Sequence
Mini-G_s C96A fw	GCCTTCAACGATGTGACTGCCATCATCTTCG
Mini-G_s C96A rv	CTGGATCCACTTGCGGCGTTCATCG
β_1AR W130E fw	GAGACCTTGTGCGTCATCG
β_1AR W130E rv	GATGCTTGCCGTCACG
β_1AR L289M fw	ATGAAGACATTGGGTATCATCATGG
β_1AR L289M rv	AGCTTTGTGTTCCCTCATGG
β_1AR N-Avi fw	GCAGAAAATTGAATGGCATGAAGGGGCCGAGCTGC
β_1AR N-Avi rv	GCTTCAAAAATATCGTTCAGGCCCATTTTGGGGGATCCGTATTTATAG

Supplementary Methods

BLI experimental procedure BLI experiments assessing β_1AR binding kinetics to binding partners were performed in triplicate, with n=3 separate tips and corresponding wells for immobilisation of receptor and mini-G protein binding, and an additional reference channel. The reference channel wells were prepared with the exact conditions of each equivalent experimental well, with the exception of the receptor loading well which contained unbiotinylated avi-β_1AR. The unbiotinylated avi-β_1AR was prepared using material separated from the biotinylated avi-β_1AR material prior to the biotinylation treatment step described in Methods.
Biosensor tips were blocked using 0.1% BSA by incubating in buffer for 10 minutes prior to experiments. BLI protocols for different experiments followed the same loading steps for receptor immobilisation. A baseline step (120 s) was recorded prior to receptor loading. Receptor loading and dissociation of any non-specifically bound material was performed as described in Methods. A baseline step was performed and an association step (300-600 s) into buffer containing agonist was performed to assess effects of ligand on BLI responses and as an additional blocking step prior to analyte association. Association and dissociation steps to assess analyte binding kinetics were performed as described in Methods, and baseline steps were performed between each new association/dissociation step. Reference channels followed the same protocols and were used to subtract any non-specific interactions between tips and analyte from the experimental data during processing. The n=3 individual binding isotherms, each generated from a different biosensor, were used to calculate binding kinetics k_{obs} and k_{off}, which then were used to obtain k_{on} and K_D values as detailed in the Methods section. Means and SDs of the n=3 individual repeats were then calculated for each kinetic parameter. A scheme and example of BLI procedures is shown in Supplementary Fig. 3a.
BLI controls Non-specific association of receptor to biosensors was assessed using reference channels with loading of unbiotinylated avi-β_1AR constructs, and the β_1AR-E construct used for NMR (no avi-tag) untreated and with biotinylation treatment. Each control showed minimal non-specific association of receptor to biosensors, and no significant loading of biotinylation treated β_1AR-E indicated minimal off-target biotinylation of receptor at Lys residues outside the avi-tag. Loading controls are shown in Supplementary Fig. 3b-c. Specific association of mini-G_s with receptor was confirmed by loading of an unrelated biotinylated protein (biotinylated avi-tagged SARS-CoV2 Spike protein Receptor Binding Domain (RBD) (kindly provided by the Hyvönen group, Dept. of Biochemistry, University of Cambridge) onto biosensors in place of receptor and recording association and dissociation curves with mini-G_s and isoprenaline (Supplementary Fig. 3d).
Concentration ranges Loading of receptor to biosensors used 300 nM receptor. Agonist concentrations used were 500 µM to ensure >99% bound receptor. Analyte concentrations ranged from 31 nM to 4000 nM. Above 4000 nM, deposition of analyte was observable on the reference channel biosensors, and thus no experimental conditions were used above this value. Concentration ranges used in BLI experiments are shown in Supplementary Table 4 and concentration trials on the reference channel are shown in Supplementary Fig. 3e.
BLI measurements and dose-responses Due to the substantially different affinities between the differing ternary complexes, different concentrations of analyte were utilised for each ternary complex, shown in Supplementary Table 4. Dose-response experiments and/or single point measurements were recorded to obtain kinetic values. Dose-response experiments were recorded with a dilution series and used to calculate K_D and R_{max} values as described in methods. Single point measurements were recorded through pre-inspection of isotherms recorded at 2-3 different concentrations and selection of the concentration which gave high-quality monophasic data with the lowest residuals. For the high affinity interactions (full agonist and canonical mini-G_s protein), both dose-response and optimised single point measurements were utilised, which gave similar results (Supplementary Fig. 4, Supplementary Table 1). Response curves were also plotted following the equation Response = (R_{max} * $\text{Concentration}_{analyte}$)/($K_D$ + $\text{Concentration}_{analyte}$).

Due to the concentration limit from deposition on biosensors described above, dose-response experiments were not performed for lower affinity interactions (non-canonical mini-G proteins, partial agonists, example of unused dose response for mini-G_{o1} shown in Supplementary Fig. 3f). Instead, single point measurements were recorded with optimised concentrations between 1 - 4 µM to obtain sufficient signal to obtain kinetic parameters and give the lowest residuals.

Assessment of analyte stability The functionality and stability of analytes was assessed by ^{1}H 1D NMR experiments and comparing the amide fingerprint regions and well separated peaks in the upfield (0 to -1 ppm) region. Samples were measured at two time points separated at least 2-fold longer than BLI experiments and incubated at 298 K between these time points. An example of mini-G_s stability assessment is shown in Supplementary Fig. 3g.

Supplementary References

1. Johnstone, E. K. M. *et al.* Investigation of Receptor Heteromers Using NanoBRET Ligand Binding. *Int. J. Mol. Sci.* **22**, 1082 (2021).
2. Barkan, K. *et al.* Pharmacological characterisation of novel adenosine A_3 receptor antagonists. *Sci. Rep.* **10**, 20781 (2020).
3. Olsen, R. H. J. *et al.* TRUPATH, an open-source biosensor platform for interrogating the GPCR transducerome. *Nat. Chem. Biol.* **16**, 841–849 (2020).
4. Liu, X. *et al.* Structural Insights into the Process of GPCR-G Protein Complex Formation. *Cell* **177**, 1243-1251 (2019).
5. Carpenter, B. & Tate, C. G. Engineering a minimal G protein to facilitate crystallisation of G protein-coupled receptors in their active conformation. *Protein Eng. Des. Sel.* **29**, 583–593 (2016).
6. London, R. E., Wingad, B. D. & Mueller, G. A. Dependence of amino acid side chain ^{13}C shifts on dihedral angle: Application to conformational analysis. *J. Am. Chem. Soc.* **130**, 11097–11105 (2008).
7. Butterfoss, G. L. *et al.* Conformational dependence of ^{13}C shielding and coupling constants for methionine methyl groups. *J. Biomol. NMR* **48**, 31–47 (2010).
8. Liu, D. & Wüthrich, K. Ring current shifts in ^{19}F-NMR of membrane proteins. *J. Biomol. NMR* **65**, 1–5 (2016).
9. Moukhametzianov, R. *et al.* Two distinct conformations of helix 6 observed in antagonist-bound structures of a β_1-adrenergic receptor. *Proc. Natl. Acad. Sci. USA* **108**, 8228–8232 (2011).
10. Su, M. *et al.* Structural Basis of the Activation of Heterotrimeric Gs-Protein by Isoproterenol-Bound β_1-Adrenergic Receptor. *Mol. Cell* **80**, 59–71 (2020).
11. Xu, X. *et al.* Binding pathway determines norepinephrine selectivity for the human β_1AR over β_2AR. *Cell Res.* **31**, 569–579 (2021).
12. Warne, T. *et al.* The structural basis for agonist and partial agonist action on a β_1-adrenergic receptor. *Nature* **469**, 241–245 (2011).
13. Imai, S. *et al.* Structural equilibrium underlying ligand-dependent activation of β_2-adrenoreceptor. *Nat. Chem. Biol.* **16**, 430–439 (2020).
14. Rasmussen, S. G. F. *et al.* Crystal structure of the β_2 adrenergic receptor–Gs protein complex. *Nature* **477**, 549–555 (2011).
15. Solt, A. S. *et al.* Insight into partial agonism by observing multiple equilibria for ligand-bound and G_s-mimetic nanobody-bound β_1-Adrenergic receptor. *Nat. Commun.* **8**, 1795 (2017).
16. Frei, J. N. *et al.* Conformational plasticity of ligand-bound and ternary GPCR complexes studied by ^{19}F NMR of the β_1-adrenergic receptor. *Nat. Commun.* **11**, 669 (2020).
17. Bundi, A. & Wuthrich, K. ^{1}H-NMR Parameters of the Common Amino Acid Residues Measured in Aqueous Solutions of the Linear Tetrapeptides H-Gly-Gly-X-L-Ala-OH. *Biopolymers* **18**, 285–297 (1979).
18. Nehmé, R. *et al.* Mini-G proteins: Novel tools for studying GPCRs in their active conformation. *PLoS ONE* **12**, e0175642 (2017).
19. Baker, J. G., Proudman, R. G. W. & Tate, C. G. The pharmacological effects of the thermostabilising (m23) mutations and intra and extracellular (β36) deletions essential for crystallisation of the turkey β-adrenoceptor. *N.-S. Arch. Pharmacol.* **384**, 71–91 (2011).
